# Assessing the need for pre-mental health competencies in undergraduate education: insights from graduate faculty surveys

**DOI:** 10.3389/fpsyg.2023.1252451

**Published:** 2024-01-05

**Authors:** Kerstin K. Blomquist, Susan J. Wenze, C. J. Eubanks Fleming, Stephanie M. Ernestus

**Affiliations:** ^1^Department of Psychology, Furman University, Greenville, SC, United States; ^2^Department of Psychology, Lafayette College, Easton, PA, United States; ^3^Department of Psychology, Elon University, Elon, NC, United States; ^4^Department of Psychology, Stonehill College, Easton, MA, United States

**Keywords:** clinical competencies, mental healthcare, undergraduate, psychology education, training

## Abstract

**Introduction:**

Despite the value of clinical competencies for masters- and doctoral-level practitioners as well as the tremendous variability in preparedness for graduate school and at graduation from graduate school, there are no competency standards for students pursuing mental healthcare careers prior to graduate study. This study aimed to identify potential pre-mental health competency standards for undergraduates pursuing mental healthcare careers.

**Methods:**

Faculty at masters and doctoral programs in a range of mental healthcare fields were asked to rate their expectations of entry-level competence and the perceived entry-level competence of their first-year, bachelor-level graduate students on 42 sub-competencies derived from the APA’s Competency Benchmarks in Professional Psychology.

**Results:**

Faculty of both masters (*N* = 320) and doctoral (*N* = 220) programs reported high expectations of first-year graduate students for 11 competency categories (professional values/attitudes; relationships; management-administration; interdisciplinary systems; individual/cultural diversity; advocacy; scientific knowledge and methods; reflective practice, self-assessment, and self-care; ethical standards and policy; supervision, and research/evaluation) and 25 sub-competencies. Faculty in masters programs rated students as not meeting their expectations in 28 sub-competencies, while faculty in doctoral programs rated students as not meeting their expectations in 17 sub-competencies. Faculty recommended internships as well as improvement in writing, counseling skills, professional behavior, diversity, equity, and inclusion, cultural competence and humility, research methods, reading research, connecting research to practice, and education about the different mental healthcare professions.

**Discussion:**

Our findings suggest that students would benefit from intentional training in multiple pre-mental health competency areas at the undergraduate level to facilitate graduate-level training in mental healthcare and to better prepare our future clinicians.

## 1 Introduction

Mental healthcare is crucial for overall health and wellbeing, yet far too many people in need of treatment do not receive it. Even prior to the onset of the COVID-19 pandemic, there was a large, world-wide, unmet need for mental healthcare (The World Mental Health Survey Consortium, 2004). This need has risen exponentially since early 2020 (Marques et al., 2020; Sanderson et al., 2020). By some estimates, over 15% of adults in the United States currently require but do not receive mental healthcare (Sammons, 2022), and the dramatic rise in emergency room visits, suicide attempts, depression, anxiety, trauma, and loneliness among youth led the American Academy of Pediatrics, the American Academy of Child and Adolescent Psychiatry, and the Children’s Hospital Association to jointly issue a declaration of a national emergency in child and adolescent mental health (American Academy of Pediatrics, 2021). Well-trained providers in all fields of mental healthcare—clinical psychologists, counselors, social workers, marriage and family therapists, school psychologists, and more—are urgently needed to fill this critical gap.

A key element in producing skilled, knowledgeable, and capable providers is establishing professional standards of care. Numerous professional organizations identify competencies pertaining to mental health knowledge and skills. The Council of University Directors of Clinical Psychology (CUDCP) has recommended pre-doctoral competencies (Council of University Directors of Clinical Psychology [CUDCP], 2021) based on the American Psychological Association (APA)’s 16 clinical competencies for doctoral students (Grus, 2014). Similarly, the Council on Social Work Education (CSWE) and the Commission on Accreditation for Marriage and Family Therapy Education (COAMFTE) have identified 9 and 5 core competencies for their fields, respectively (Council on Social Work Education [CSWE], 2015; Commission on Accreditation for Marriage and Family Therapy Education [COAMFTE], 2021). Further, the APA generated a list of skills to be acquired with a bachelor-level psychology major (Naufel et al., 2018) alongside guidelines for the undergraduate psychology major (American Psychological Association [APA], 2022). However, these skills and guidelines are general to all types of employment without a focus on attending graduate school in a helping area.

Amidst these diverse sets of guidelines, expectations for foundational provider competencies that are relevant to all mental health fields and achievable during undergraduate education are conspicuously lacking. This is striking for several reasons. First, other professional healthcare education organizations, such as the Association of American Medical Colleges (AAMC), have identified core competencies for matriculating students (American Association of Medical Colleges [AAMC], 2022); “pre-health” or “pre-med” tracks are common at the undergraduate level to ensure that students meet these expectations via required coursework and standardized clinical experiences. Second, “pre-mental health” competency standards would be beneficial even for those who do not pursue graduate study in the field of mental health, as the helping professions increasingly rely on bachelor-level employees to provide mental health services. For example, task-sharing models (Raviola et al., 2019), specialized certificate programs (Hubbard, 2022), and mental health navigators (Godoy et al., 2019) all show great promise for successfully training and employing non-specialist mental healthcare workers to provide urgently-needed services amid unprecedented mental health needs. Recent data indicate that, of approximately 1.5 million Americans in the workforce whose highest degree is a bachelors in psychology, 201,000 (13.4%) are employed in healthcare occupations, with 112,700 (7.5%) employed as bachelor-level counselors or social workers (American Psychological Association [APA], 2021). Unfortunately, research on healthcare employers’ expectations regarding skillsets in bachelor-level providers is lacking, but even in non-healthcare fields (e.g., business; Landrum and Harrold, 2003), abilities such as listening skills, interpersonal relationship skills, ethical decision-making, and understanding human behavior are deemed very important in job applicants who hold a BA in psychology. Foundational provider competency guidelines could also help replace biased assessments like the GRE and level the playing field between privileged students—who know to seek out and are able to acquire internships, practica, research placements, and other training experiences—and other students who lack awareness of or the resources to adhere to this “hidden curriculum” (De Los Reyes and Uddin, 2021). Finally, establishing common standards at the undergraduate level alongside steps to support equitable access to achieve these standards could improve performance in graduate school and in the workplace; facilitate graduate admissions decisions; streamline graduate education; assist in employment; improve delivery of evidence-based practice; promote interdisciplinary dialogue and care; and better prepare individuals to receive care (Kathol et al., 2010; Cubic et al., 2012; Ernestus et al., 2022).

A first step in formulating practical, pertinent, and measurable undergraduate “pre-mental health” competency standards is to survey key stakeholders about what competencies they expect undergraduate students to have and how well students meet those expectations. Faculty in graduate mental healthcare programs work directly with bachelor-level first-year graduate students to prepare them to be mental healthcare providers and are important stakeholders in identifying the skills needed to become successful providers. It is important to note that, at the undergraduate level, students are not typically siloed into a specific mental healthcare discipline; therefore, graduate faculty in a range of mental healthcare programs are stakeholders for pre-mental health competency standards. That is, regardless of a specific discipline’s approach to providing mental healthcare, we propose that there are foundational provider competencies relevant to all fields of mental healthcare that can be specified for undergraduates. Further, in order to reduce the siloing of different mental healthcare fields, to improve collaborative care and intervention outcomes, and to reflect the nature of undergraduate education, we employ the terms “clinical” and “clinical competencies” interchangeably with “pre-mental health” and “pre-mental health competencies” to refer broadly and inclusively to any mental healthcare-related discipline and skill. Moreover, some undergraduates pursue masters-level degrees and some pursue doctoral-level degrees within mental healthcare-related fields; however, similar to different disciplines, we propose that there are foundational provider competencies common to different degree levels.

The current cross-sectional study assessed graduate faculty expectations of entry-level competence and perceptions of actual entry-level competence among bachelor-level, incoming masters and doctoral students across an array of potential practice-related domains, and in a diverse range of mental healthcare-related graduate programs. We specifically assessed graduate-level faculty because they can tell us what they need in incoming graduate students. Faculty were also asked about recommended experiences at the undergraduate level that would better prepare students for graduate study or employment in mental health-related fields. The competency areas assessed were based on the APA’s Competency Benchmarks in Professional Psychology (American Psychological Association [APA], 2012), which include standards for determining readiness for practicum, internship, and professional practice. In sum, the study aimed to answer the following questions: (Q1) Which clinical competencies do graduate faculty expect first-year graduate students with bachelor degrees to have? (Q2) Do expectations differ between faculty teaching in masters- and doctoral-level programs? (Q3) Are there discrepancies between expected competencies and perceived actual competence for incoming graduate students in mental health-related programs, as rated by graduate faculty, and what is the magnitude of discrepancy? (Q4) Does perceived actual competence differ between faculty teaching in masters- and doctoral-level programs? (Q5) What insights can graduate faculty provide about how to better prepare undergraduates for graduate study and employment as a future mental healthcare provider?

## 2 Materials and methods

### 2.1 Programs identified

To procure a representative sample of mental health-related masters and doctoral programs in the United States for this cross-sectional study, programs were identified via accreditation websites (see Supplementary Table 1) as well as via online searches by state, and word-of-mouth. Mental health programs were defined broadly and included clinical psychology, counseling, social work, marriage and family therapy, school counseling, school psychology, addiction counseling, and clinical rehabilitation counseling. Online searches involved entering different program types (e.g., “counseling masters program”), masters vs. doctoral degrees (e.g., “MA,” “MEd,” or “PhD”), and states until all program type by state combinations were exhausted. There were 802 masters programs identified: 16.5% from the West, 25.9% from the Midwest, 36.1% from the South and 21.3% from the Northeast. The majority of the masters programs identified were in the field of social work (34.7%), clinical mental health counseling (27.8%), school counseling (18.0%), and marriage and family therapy (8.1%). There were 374 doctoral programs identified: 21.9% from the West, 22.7% from the Midwest, 33.2% from the South, and 22.2% from the Northeast. The majority of the doctoral programs identified were in the field of clinical (55.6%), counseling (18.2%), and school psychology (16.3%).

### 2.2 Faculty identified

Faculty teaching in these programs were identified via program websites. A total of 7,741 faculty were identified from the masters programs with 15.7% of faculty from the West, 26.6% of faculty from the Midwest, 37.2% of faculty from the South, and 20.5% of faculty from the Northeast. The majority of the faculty identified from masters programs were in the field of social work (59%), clinical mental health counseling (19.4%), school counseling (9.2%), and marriage and family therapy (3.9%). From the doctoral programs, 3,726 faculty were identified with 21.8% of faculty from the West, 19.0% of faculty from the Midwest, 33.4% of faculty from the South, and 26.1% of faculty from the Northeast. The majority of the faculty identified from doctoral programs were in the field of clinical (64.6%), counseling (15.7%), and school psychology (9.9%). As it was not possible to determine which faculty taught first-year graduate students by looking at program websites, faculty of all positions/titles were identified (e.g., affiliate, visiting, adjunct, instructors, assistant professors, etc.). When it was unclear whether a person listed on the website would be eligible, they were included.

### 2.3 Participants

Inclusion criteria were faculty at a masters or doctoral-level graduate mental healthcare-related program who taught first-year graduate students. Faculty who did not teach in a mental healthcare-related program and/or did not teach first-year graduate students were not eligible to participate.

**FIGURE 1:**
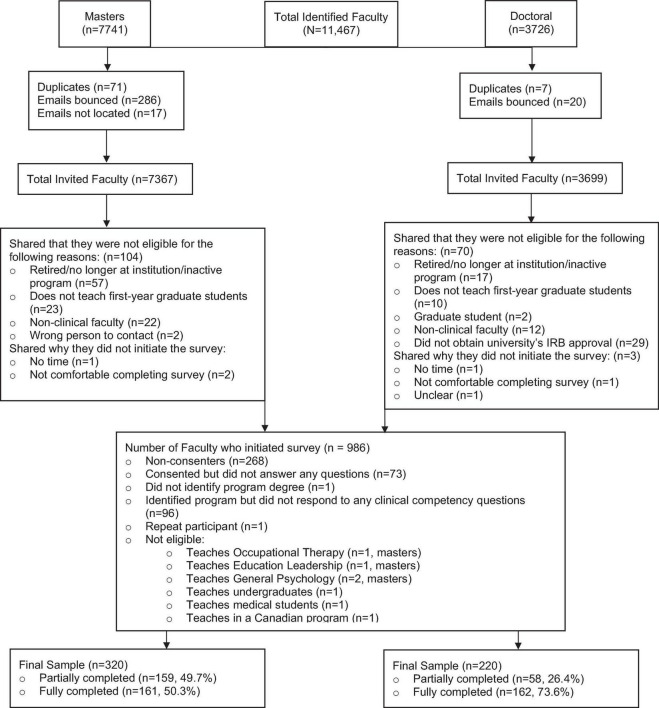
CONSORT flow diagram.

All 11,066 faculty identified received an invitation to participate in our survey. Nine hundred and eighty-six participants initiated the survey. Several participants were excluded from analyses because they were not faculty at programs in the United States or were not in a mental-health related field (see Figure 1 Consort Flow Chart). Our final sample comprised 540 participants: 320 faculty teaching in masters programs and 220 faculty teaching in doctoral programs. Thus, 4.8% of the faculty who received a survey invitation and 54.8% of those who initiated the survey met inclusion criteria and completed at least one of the clinical competency survey questions. Of the 540 participants included in the final sample, 50% of faculty in masters programs and 74% of faculty in doctoral programs completed the survey. Median study completion time was 18.6 min.

### 2.4 Survey

The survey was administered through Qualtrics (Qualtrics Inc., Provo, Utah). Participants were asked to first share the name of the program they teach in, the degree awarded to students in that program, and the program’s field of mental healthcare (e.g., social work, school counseling, school psychology, clinical psychology, etc.). If participants taught in more than one program, they were asked to select only one program and to preferentially select a masters program over a doctoral program. Participants were instructed to respond to the remaining questions based on the program and field they indicated at the beginning of the survey (see Supplementary Appendix A for survey). In addition, participants were instructed to “rate your average first-year graduate student” and to “rate only those with bachelor degrees–not those with additional graduate experience.”

The survey then asked participants to share (1) their expectations regarding clinical competencies of their first-year graduate students with bachelor degrees and (2) the perceived actual entry-level of competence of their first-year graduate students with bachelor degrees in fourteen overarching clinical competency areas composed of 42 sub-competencies. Participants rated expectations on a scale from 0 (do not expect competence) to 10 (expect high competence) and rated entry-level of competence on a scale from 0 (no competence) to 10 (extremely competent). Provider competencies were derived directly from the APA’s Competency Benchmarks in Professional Psychology,^1^ which include sixteen overarching areas of competencies. Based on our experience teaching undergraduates and the perceived relevance to undergraduate training and incoming first-year graduate students, we selected fourteen of the 16 competency areas composed of 40 (out of 55) sub-competencies that we believed to be achievable by undergraduate students by the time of their graduation (see Table 2). For example, consultation and teaching competencies were not included because they were deemed not relevant for undergraduate students. We further revised the description of the sub-competencies to be appropriate at the undergraduate level instead of using the descriptions provided for the “readiness for practicum,” “readiness for internship,” or “readiness for entry to practice” levels. In addition, participants were asked to rate their expectations and the perceived entry-level of competence of students in “reading, understanding, and interpreting clinical research,” specifically randomized controlled trials (RCTs) and meta-analyses as these can be taught at the undergraduate level and are not directly assessed by the APA’s competencies, resulting in 15 overarching competencies (42 sub-competencies) evaluated.

Participants were further asked to indicate the number of years they had taught in the program listed, their highest awarded degree, what they would recommend to undergraduate psychology departments to better prepare students to engage in mental healthcare careers as well as what courses, topics, and skills they would recommend for this purpose.

### 2.5 Procedure

Furman University’s Internal Review Board approved this study. Participants were invited to participate in the survey via email and were told that the purpose of the study was to “explore clinical competency standards for undergraduate students pursuing a career in mental healthcare in order to develop mental healthcare-related curriculum at the undergraduate level” (see Supplementary Appendix A for outreach email). Participants received at least one reminder to complete the survey. All participants provided informed written consent before completing the survey. After the survey, participants could enter a raffle to win one of eight $50 cash awards.

### 2.6 Statistical analyses

To analyze our data, we conducted a series of *t*-tests with continuous variables and chi-squares with categorical variables. Due to multiple comparisons, the *p*-value was set at *p* ≤ 0.001. Cohen’s *d* was computed for all *t*-tests; 0.2 is a small effect, 0.5 is a medium effect, and 0.8 is considered a large effect (Cohen, 1988). All statistical analyses were conducted using the IBM Statistical Package for the Social Sciences (SPSS) version 27.

More specifically, to answer our first question (Q1), we calculated the mean expectation and perceived entry-level of competence for each of the fifteen overarching competency areas by averaging together the sub-competencies comprising each area together. Expectations were scored on an 11-point scale from 0 to 10, where 5 is the midpoint and 6 is above the midpoint and just shy of the top tertile. A minimum expectation score of 6.0 was set; mean expectation scores of 6.0 and above were deemed to reflect moderate to high expectations among graduate faculty. If the mean expectation score for either masters or doctoral programs met this minimum expectation score, the competency was included.

To answer our second and fourth questions (Q2 and Q4), independent samples *t*-tests were conducted to compare masters programs to doctoral programs on expectations and perceived entry-level of student competence.

To answer our third question (Q3), paired samples *t*-tests were conducted to compare expectations and perceived entry-level of competence (separately for masters and doctoral programs).

To answer our fifth question regarding faculty recommendations (Q5), inductive qualitative coding analysis was employed, which allows for themes to emerge (Bingham and Witkowsky, 2022; Vears and Gillam, 2022). We chose this method instead of deductive qualitative analysis because our research is exploratory versus based on a specific theory (Vears and Gillam, 2022). Open-ended written responses to the question “What would you recommend to undergraduate psychology departments to better prepare students to engage in their mental healthcare careers?” were openly coded by KKB allowing codes to emerge separately for masters and doctoral programs. CJF also independently and openly coded 25% of the data. Then coding patterns across responses were identified and grouped into themes. Themes reported by fewer than two participants were removed. Reliability between KKB and CJF was calculated using Krippendorff’s alpha (Krippendorff, 2004). Krippendorff’s alpha is considered to be acceptable at 0.66, good at 0.8, and perfect at 1. Given low numbers of codes for some items, a few initial alphas were below the 0.8 cutoff. Discrepancies between raters were discussed until agreement was reached and then responses were recoded, after which all alphas were in the good range (≥0.8). Our percent agreement was 92% or higher for all items. The frequency of each theme was computed separately for masters and doctoral programs.

## 3 Results

### 3.1 Program and faculty characteristics

Table 1 presents the program and faculty characteristics. Of the 540 participants, 320 were faculty representing 229 masters programs, and 220 were faculty representing 129 doctoral programs. The majority of masters programs awarded MSW (52%), MA (24%), and MS (11%) degrees. The majority of doctoral programs awarded PhD (68%) and PsyD (30%) degrees. Social work (50%) and counseling (29%) were the fields most represented by masters programs whereas clinical (75%), counseling (11%), and school psychology (10%) were the fields most represented by doctoral programs. The majority of faculty reported having taught in their respective programs for six or more years (63% for masters-program faculty; 69% for doctoral-program faculty). The majority of the faculty in both masters and doctoral programs had earned a PhD (73% for masters-program faculty; 90% for doctoral-program faculty).

**TABLE 1:** Program and faculty characteristics.

	Masters (*N* = 320)	Doctoral (*N* = 220)	Masters vs. Doctoral
Number of programs identified	229^a^	135^a^	
**Program degree**	**n (%)**	**n (%)**	
MA	77 (24.1)		540.00 (9), <0.001
MS	35 (10.9)		
MEd	21 (6.6)		
MSW	167 (52.2)		
MFT	7 (2.2)		
EdS	10 (3.1)		
DSW		2 (0.9)	
PsyD		68 (30.9)	
PhD		150 (68.2)	
Other (e.g., MDiv, Graduate Certificate, SSP)	3 (0.9)		
**Program field**	**n (%)**	**n (%)**	
Clinical	20 (6.3)	165 (75.0)	326.28 (8), <0.001
Counseling	92 (28.7)	24 (10.9)	
School psychology	14 (4.4)	21 (9.5)	
Social work	161 (50.3)	4 (1.8)	
School counseling	5 (1.6)		
Marriage and family therapy	22 (6.9)	1 (0.5)	
Clinical and counseling	2 (0.6)	1 (0.5)	
Clinical and school psychology		3 (1.4)	
Other (Applied Behavior Analysis, Applied Psychology, Creative Arts Therapy, Child Life, Intellectual and Developmental Disabilities)	4 (1.3)	1 (0.5)	
**Years faculty taught in program**	**n (%)**	**n (%)**	***X^2^ (df), p***
1–2 years	40 (12.5)	24 (10.9)	2.32 (2), 0.313
3–5 years	79 (24.7)	44 (20.0)	
6 + years	201 (62.8)	152 (69.1)	
**Highest degree faculty earned**	**n (%)**	**n (%)**	
MA	4 (1.3)		56.46 (8), <0.001
MS	2 (0.6)	1 (0.5)	
MSW	45 (14.1)		
MEd	4 (1.3)		
EdD	10 (3.1)	3 (1.4)	
DSW	9 (2.8)		
PsyD	10 (3.1)	18 (8.2)	
PhD	232 (72.5)	197 (89.5)	
Other (MD, DMin, PharmD, Post-Grad Certificate)	4 (1.3)	1 (0.5)	

^a^There were missing data for 8 masters-level programs and 32 doctoral-level programs, so there may have been more programs represented than are accounted for in these numbers.

**TABLE 2:** Faculty expectations, perceived level of competence of first-year graduate students, and potential undergraduate clinical competencies.

	Masters			Doctoral			
	Expectations *M(SD)*	Competence *M(SD)*	E vs. C *t(df), p, d*	Expectations *M(SD)*	Competence *M(SD)*	E vs. C *t(df), p, d*	Masters vs. Doctoral *t(df), p, d*
*PROFESSIONAL VALUES AND ATTITUDES: “behavior and comportment that reflect the values and attitudes of psychology”	**8.67 (1.62)**	**7.49 (1.64)**	**12.73 (312)** ***p* < 0.001, *d* = 0.72**	**8.78 (1.32)**	**7.84 (1.42)**	**12.36 (215)** ***p* < 0.001, *d* = 0.84**	**Exp:**−**1.03 (524.9), 0.304,**−**0.09** **Comp:**−**2.58 (527), 0.010,**−**0.23**
*Integrity: “honesty, personal responsibility and adherence to professional values”	9.19 (1.93)	7.91 (1.88)	12.83 (311) *p* < 0.001, *d* = 0.73	9.58 (1.52)	8.42 (1.78)	12.45 (215) *p* < 0.001, *d* = 0.85	Exp: −2.50 (526.4), 0.013, −0.21 Comp: −3.05 (526), 0.002, −0.27
*Deportment: “conducting oneself in a professional manner”	8.84 (1.88)	7.38 (1.89)	13.21 (311) *p* < 0.001, *d* = 0.75	8.79 (1.80)	7.66 (1.74)	10.01 (213) *p* < 0.001, *d* = 0.68	Exp: 0.62 (534), 0.535, 0.06 Comp: −1.54 (524), 0.125, −0.14
*Accountability: “accountable and reliable”	9.27 (1.60)	7.61 (1.82)	14.95 (307) *p* < 0.001, *d* = 0.85	9.64 (1.42)	8.14 (1.64)	15.74 (215) *p* < 0.001, *d* = 1.07	Exp: −2.76 (533), 0.006, −0.24 Comp: −3.24 (522), 0.001, −0.29
*Concern for the Welfare of Others: “demonstrating awareness of the need to uphold and protect the welfare of others”	9.50 (1.84)	8.61 (2.01)	9.26 (310) *p* < 0.001, *d* = 0.53	9.75 (1.44)	9.10 (1.64)	8.02 (215) *p* < 0.001, *d* = 0.55	Exp: −1.75 (527.7), 0.081, −0.15 Comp: −3.08 (509.3), 0.002-0.26
*Professional Identity: “demonstrating an understanding of self as professional; thinking like a clinician”	6.42 (2.68)	5.83 (2.34)	4.63 (310) *p* < 0.001, *d* = 0.26	6.15 (2.48)	5.87 (2.08)	2.17 (214) *p*** = 0**.031, *d* = 0.15	Exp: 1.05 (533), 0.295, 0.09 Comp: −0.23 (524), 0.819, −0.02
*INDIVIDUAL AND CULTURAL DIVERSITY: “awareness, sensitivity and skills in working professionally with diverse individuals, groups, and communities who represent various cultural and personal background and characteristics defined broadly and consistent with APA policy”	**6.83 (2.21)**	**5.92 (2.14)**	**7.50 (289)** ***p* < 0.001, *d* = 0.44**	**6.42 (1.89)**	**6.06 (1.64)**	**3.46 (202)** ***p* < 0.001, *d* = 0.24**	**Exp: 2.01 (480.5), 0.045, 0.18** **Comp:**−**0.85 (486.8), 0.398,**−**0.07**
*Self as Shaped by Cultural Diversity: “demonstrating knowledge, awareness, and understanding of one’s own dimensions of diversity and attitudes toward diverse others”	7.16 (2.24)	6.20 (2.14)	7.48 (289) *p* < 0.001, *d* = 0.44	6.90 (1.91)	6.55 (1.68)	2.91 (202) *p* = 0.004, *d* = 0.20	Exp: 1.26 (481.1), 0.208, 0.11 Comp: −1.99 (484.5), 0.047, −0.18
*Others as Shaped by Cultural Diversity: “demonstrating knowledge, awareness, and understanding of other individuals as cultural beings”	7.20 (2.19)	6.28 (2.19)	7.25 (289) *p* < 0.001, *d* = 0.43	6.87 (1.85)	6.54 (1.64)	3.06 (202) *p* = 0.003, *d* = 0.22	Exp: 1.63 (482.9), 0.105, 0.14 Comp: −1.50 (488.9), 0.134, −0.13
*Interaction of Self and Others as Shaped by Cultural Diversity and Context: “demonstrating knowledge, awareness, and understanding of interactions between self and diverse others”	6.98 (2.25)	6.00 (2.21)	7.72 (287) *p* < 0.001, *d* = 0.46	6.51 (2.02)	5.98 (1.79)	4.96 (202) *p* < 0.001, *d* = 0.35	Exp: 2.19 (495), 0.029, 0.20 Comp: 0.08 (480.6), 0.937, 0.01
Applications based on Cultural Context: “demonstrating knowledge of and sensitivity to the scientific, theoretical, and contextual issues related to individual and cultural diversity as they apply to assessment, treatment, research, relationships, etc.”	5.95 (2.65)	5.18 (2.44)	5.85 (287) *p* < 0.001, *d* = 0.35	5.39 (2.43)	5.19 (2.16)	1.58 (202) *p* = 0.115, *d* = 0.11	Exp: 2.15 (496), 0.032, 0.20 Comp: −0.07 (464.7), 0.043, −0.01
*ETHICAL LEGAL STANDARDS AND POLICY: “application of ethical concepts and awareness of legal issues regarding professional activities with individuals, groups, and organizations”	**6.04 (2.72)**	**5.63 (2.52)**	**2.87 (270)** ***p* = 0.004, *d* = 0.18**	**6.04 (2.13)**	**5.80 (2.05)**	**2.55 (190)** ***p* = 0.012, *d* = 0.19**	**Exp:**−**0.08 (462.4), 0.939,**−**0.01** **Comp:**−**0.78 (450.6), 0.434,**−**0.07**
Knowledge of ethical, legal and professional standards and guidelines: “demonstrating knowledge of the principles of the APA Ethical Principles and Code of Conduct”	5.14 (3.37)	5.04 (3.14)	0.58 (268) *p* = 0.566, *d* = 0.04	5.03 (2.52)	5.01 (2.40)	0.15 (189) *p* = 0.878, *d* = 0.01	Exp: 0.30 (465.5), 0.764, 0.03 Comp: 0.13 (454.3), 0.895, 0.01
Awareness and Application of Ethical Decision Making: “demonstrating awareness of the importance of applying an ethical decision model to practice”	5.44 (3.01)	5.01 (2.81)	2.68 (269) *p* = 0.008, *d* = 0.16	5.14 (2.74)	5.04 (2.65)	0.85 (190) *p* = 0.398, *d* = 0.06	Exp: 0.91 (467), 0.363, 0.09 Comp: −0.12 (459), 0.906, −0.01
*Ethical Conduct: “displaying ethical attitudes and values”	7.60 (2.71)	6.85 (2.54)	5.55 (270) *p* < 0.001, *d* = 0.34	7.98 (2.18)	7.37 (2.10)	6.32 (190) *p* < 0.001, *d* = 0.46	Exp: −1.68 (459.7), 0.094, −0.15 Comp: −2.41 (448.2), 0.017, −0.22
*REFLECTIVE PRACTICE, SELF-ASSESSMENT, SELF-CARE “practice conducted with personal and professional self-awareness and reflection; with awareness of competencies; with appropriate self-care”	**6.55 (2.43)**	**5.80 (2.31)**	**5.52 (270)** ***p* < 0.001, *d* = 0.34**	**6.22 (2.08)**	**5.76 (2.00)**	**4.37 (190)** ***p* < 0.001, *d* = 0.32**	**Exp: 1.49 (451.1), 0.137, 0.14** **Comp: 0.19 (441.5), 0.847, 0.02**
*Reflective Practice: “Displaying mindfulness and self-awareness; engaging in reflection regarding professional practice”	6.66 (2.61)	5.75 (2.51)	6.01 (268) *p* < 0.001, *d* = 0.37	5.79 (2.59)	5.46 (2.43)	2.56 (190) *p* = 0.011, *d* = 0.19	Exp: 3.36 (466), < 0.001, 0.32 Comp: 1.26 (458), 0.208, 0.12
Self-Assessment: “Demonstrating knowledge of core competencies; engaging in initial self-assessment re: competencies”	5.82 (2.96)	5.28 (2.72)	3.33 (267) *p* < 0.001, *d* = 0.20	5.08 (2.74)	4.85 (2.52)	1.61 (189) *p* = 0.109, *d* = 0.12	Exp: 2.61 (464), 0.009, 0.25 Comp: 1.66 (457), 0.098, 0.16
*Self-Care: “attention to personal health and wellbeing to assure effective professional functioning”	6.90 (2.35)	6.10 (2.36)	5.26 (270) *p* < 0.001, *d* = 0.32	6.59 (2.11)	6.00 (1.93)	4.28 (190) *p* < 0.001, *d* = 0.31	Exp: 1.49 (467), 0.136, 0.14 Comp: 0.50 (450.2), 0.619, 0.05
*Participation in Supervision Process: “demonstrating straightforward, truthful, and respective communication in supervisory relationship”	6.83 (3.13)	6.09 (2.99)	4.54 (270) *p* < 0.001, *d* = 0.28	7.41 (2.85)	6.74 (2.77)	4.56 (189) *p* < 0.001, *d* = 0.33	Exp: −2.07 (467), 0.039, −0.19 Comp: −2.37 (459), 0.018, −0.22
*RELATIONSHIPS “relating effectively and meaningfully with individuals, groups, and/or communities”	**8.14 (1.93)**	**6.79 (1.80)**	**11.64 (264)** ***p* < 0.001, *d* = 0.72**	**8.21 (1.61)**	**6.90 (1.63)**	**11.59 (189)** ***p* < 0.001, *d* = 0.84**	**Exp:**−**0.47 (449.0), 0.635,**−**0.04** **Comp:**−**0.68 (453), 0.499,**−**0.06**
*Interpersonal Relationships: “displaying interpersonal skills; forming and maintaining productive and respectful relationships with clients, peers/colleagues, supervisors”	8.41 (2.06)	7.33 (1.94)	10.00 (264) *p* < 0.001, *d* = 0.61	8.69 (1.74)	7.52 (1.83)	9.70 (189) *p* < 0.001, *d* = 0.70	Exp: −1.70 (447.9), 0.090, −0.16 Comp: −1.07 (453), 0.285, −0.10
*Affective Skills: “displaying affective skills; Negotiating differences and handling conflict; providing effective feedback to others and receiving feedback non-defensively”	7.70 (2.23)	6.32 (2.10)	10.37 (264) *p* < 0.001, *d* = 0.64	7.75 (1.87)	6.40 (1.85)	10.26 (188) *p* < 0.001, *d* = 0.75	Exp: −0.35 (445.9), 0.724, −0.03 Comp: −0.38 (452), 0.704, −0.04
*Expressive Skills: “communicates ideas, feelings, and information clearly using verbal, non-verbal, and written skills”	8.31 (2.02)	6.71 (1.86)	11.76 (264) *p* < 0.001, *d* = 0.72	8.19 (1.81)	6.78 (1.79)	9.95 (188) *p* < 0.001, *d* = 0.72	Exp: 0.68 (459), 0.494, 0.07 Comp: = 0.37 (452), 0.712, −0.04
*SCIENTIFIC KNOWLEDGE AND METHODS “understanding of research, research methodology, techniques of data collection and analysis, biological bases of behavior, cognitive affective bases of behavior, and development across the lifespan. Respect for scientifically derived knowledge.”	**6.53 (2.57)**	**5.62 (2.40)**	**7.56 (263)** ***p* < 0.001, *d* = 0.47**	**7.40 (1.93)**	**6.19 (2.02)**	**10.59 (187)** ***p* < 0.001, *d* = 0.77**	**Exp:**−**4.15 (455.5), < 0.001,**−**0.38** **Comp:**−**2.74 (437.6), 0.006,**−**0.25**
*Scientific Mindedness: “displaying critical scientific thinking; Valuing and applying scientific methods to professional practice	6.55 (2.63)	5.41 (2.46)	8.43 (263) *p* < 0.001, *d* = 0.52	7.55 (2.16)	6.21 (2.17)	10.43 (187) *p* < 0.001, *d* = 0.76	Exp: −4.51 (448.0), < 0.001, −0.41 Comp: −3.69 (429.7), < 0.001, −0.35
*Scientific Foundation of Psychology: “demonstrating an understanding of psychology as a science”	6.85 (3.00)	6.11 (2.83)	5.36 (260) *p* < 0.001, *d* = 0.33	8.03 (2.01)	6.73 (2.20)	10.48 (187) *p* < 0.001, *d* = 0.76	Exp: −5.11 (454.6), < 0.001, −0.45 Comp: −2.64 (444.2), 0.009, −0.24
*Scientific Foundation of Professional Practice: “understanding the scientific foundation of professional practice”	6.23 (2.82)	5.37 (2.60)	6.50 (262) *p* < 0.001, *d* = 0.40	6.63 (2.50)	5.63 (2.35)	7.40 (187) *p* < 0.001, *d* = 0.54	Exp: −1.61 (457), 0.107, −0.15 Comp: −1.12 (449), 0.262, −0.11
*RESEARCH/EVALUATION “generating research that contributes to the professional knowledge base and/or evaluates the effectiveness of various professional activities”	**5.85 (2.65)**	**5.00 (2.46)**	**5.87 (261)** ***p* < 0.001, *d* = 0.36**	**6.50 (2.17)**	**5.46 (2.20)**	**8.41 (184)** ***p* < 0.001, *d* = 0.62**	**Exp:**−**2.78 (442.3), 0.006,**−**0.26** **Comp:**−**2.01 (445), 0.045 m**−**0.19**
*Scientific Approach to Knowledge Generation: “demonstrating skills and habits in seeking, applying, and evaluating theoretical and research knowledge relevant to the practice of psychology”	5.98 (2.77)	5.15 (2.59)	5.53 (260) *p* < 0.001, *d* = 0.34	6.80 (2.24)	5.70 (2.21)	8.33 (184) *p* < 0.001, *d* = 0.61	Exp: −3.39 (443.7), < 0.001, −0.31 Comp: −2.38 (428.7), 0.018, −0.22
*Application of Scientific Method to Practice: “demonstrating knowledge of application of scientific methods to evaluating practices, interventions, and programs”	5.73 (2.69)	4.86 (2.50)	5.92 (260) *p* < 0.001, *d* = 0.37	6.19 (2.42)	5.22 (2.44)	7.65 (184) *p* < 0.001, *d* = 0.56	Exp: −1.92 (428.3), 0.055, −0.18 Comp: −1.53 (444), 0.128, −0.15
**EVIDENCE-BASED PRACTICE** “integration of research and clinical expertise in the context of patient factors”	**5.23 (2.93)**	**4.67 (2.65)**	**3.80 (259)** ***p* < 0.001, *d* = 0.24**	**5.10 (2.65)**	**4.68 (2.46)**	**3.59 (184)** ***p* < 0.001, *d* = 0.26**	**Exp: 0.47 (451), 0.637, 0.05** **Comp:**−**0.06 (443), 0.949,**−**0.01**
Knowledge and Application of Evidence-Based Practice: “demonstrating knowledge of scientific, theoretical, and contextual bases of assessment and intervention; demonstrating knowledge of the value of evidence-based practice”	5.23 (2.93)	4.67 (2.65)	3.80 (259) *p* < 0.001, *d* = 0.24	5.10 (2.65)	4.68 (2.46)	3.59 (184) *p* < 0.001, *d* = 0.26	Exp: 0.47 (451), 0.637, 0.05 Comp: −0.06 (443), 0.949, −0.01
*^a^* **READING, UNDERSTANDING, AND INTERPRETING CLINICAL RESEARCH**	**3.78 (2.94)**	**3.57 (2.88)**	**1.20 (247)** ***p* = 0.232, *d* = 0.08**	**4.45 (2.54)**	**4.02 (2.48)**	**2.87 (175)** ***p* = 0.005, *d* = 0.22**	**Exp:**−**2.62 (411.2), 0.009,**−**0.25** **Comp:**−**1.67 (422), 0.095,**−**0.17**
Randomized Controlled Trials: “Demonstrating an understanding of study design, methodological limitations, statistical interpretation, and clinical implications of findings of RCTs.”	4.02 (3.11)	3.73 (2.99)	1.61 (247) *p* = 0.108, *d* = 0.10	4.85 (2.67)	4.39 (2.61)	3.02 (175) *p* = 0.003, *d* = 0.23	Exp: −3.05 (411.5), 0.002, −0.29 Comp: −2.34 (422), 0.020, −0.23
Meta-Analyses: “Demonstrating an understanding of study design, methodological limitations, statistical interpretation, and clinical implications of findings of meta-analyses.”	3.55 (2.92)	3.39 (2.94)	0.86 (246) *p* = 0.390, *d* = 0.06	4.05 (2.62)	3.65 (2.61)	2.31 (175) *p* = 0.022, *d* = 0.17	Exp: −1.93 (429), 0.054, −0.19 Comp: −0.92 (421), 0.357, −0.09
**ASSESSMENT** “assessment and diagnosis of problems, capabilities, and issues associated with individuals, groups, and/or communities”	**3.91 (2.74)**	**3.80 (2.53)**	**0.75 (251)** ***p* = 0.454, *d* = 0.05**	**3.70 (2.18)**	**3.69 (2.25)**	**0.06 (175)** ***p* = 0.954, *d* = 0.00**	**Exp: 0.48 (421.9), 0.634, 0.05** **Comp: 0.46 (402.4), 0.648, 0.04**
Knowledge of Measurement and Psychometrics: “Demonstrating knowledge of the scientific, theoretical, and contextual basis of test construction and interviewing”	4.02 (2.96)	3.83 (2.84)	1.16 (250) *p* = 0.247, *d* = 0.07	4.24 (2.65)	3.88 (2.51)	2.35 (175) *p* = 0.020, *d* = 0.18	Exp: −0.99 (436), 0.323, −0.10 Comp: −0.18 (402.7), 0.859, −0.02
Knowledge of Assessment Methods: “Demonstrating knowledge of administration and assessment scoring, including clinical interviewing and mental status exam”	3.68 (2.87)	3.62 (2.74)	0.36 (247) *p* = 0.719, *d* = 0.02	3.49 (2.56)	3.54 (2.57)	−0.39 (174) *p* = 0.698, *d* = −0.03	Exp: 0.33 (435), 0.740, 0.03 Comp: 0.28 (421), 0.779, 0.03
Application of Assessment Methods: “Demonstrating knowledge of measurement across domains of functioning and practice settings	3.61 (2.85)	3.72 (2.84)	−0.60 (250) *p* = 0.548, *d* = −0.04	3.36 (2.54)	3.47 (2.70)	−0.71 (174) *p* = 0.478, *d* = −0.05	Exp: 0.56 (402.3), 0.574, 0.05 Comp: 0.89 (424), 0.376, 0.09
Diagnosis: “Demonstrating knowledge regarding the range of normal/abnormal behavior in the context of human development and diversity”	4.66 (2.95)	4.38 (2.60)	1.83 (251) *p* = 0.068, *d* = 0.12	5.04 (2.33)	4.80 (2.26)	1.78 (175) *p* = 0.076, *d* = 0.13	Exp: −1.79 (424.7), 0.074, −0.17 Comp: −1.78 (406.7), 0.076, −0.17
Conceptualization: “Demonstrating knowledge of formulating diagnosis and case conceptualization”	3.70 (3.00)	3.60 (2.78)	0.69 (249) *p* = 0.490, *d* = 0.04	3.33 (2.41)	3.40 (2.42)	−0.48 (174) *p* = 0.632, *d* = −0.04	Exp: 1.14 (421.2), 0.255, 0.11 Comp: 0.77 (403.2), 0.440, 0.07
Communication of Assessment Findings: “Demonstrating awareness of models of report writing and progress notes”	3.80 (3.11)	3.65 (2.85)	0.96 (249) *p* = 0.340, *d* = 0.06	2.79 (2.39)	3.02 (2.64)	−1.38 (174) *p* = 0.169, *d* = −0.10	Exp: 3.30 (426.5), 0.001, 0.31 Comp: 2.35 (391.6), 0.019, 0.23
**INTERVENTION** “interventions designed to alleviate suffering and to promote health and wellbeing of individuals, groups, and/or communities.”	**4.63 (2.82)**	**4.49 (2.66)**	**0.92 (250)** ***p* = 0.360, *d* = 0.06**	**4.10 (2.26)**	**4.18 (2.44)**	−**0.58 (175)** ***p* = 0.566, *d* =** −**0.04**	**Exp: 1.83 (420.4), 0.068, 0.17** **Comp: 1.27 (395.1), 0.205, 0.12**
Intervention Planning: “understanding the relationship between assessment and intervention”	4.27 (3.25)	4.06 (2.99)	1.21 (248) *p* = 0.226, *d* = 0.08	3.53 (2.57)	3.52 (2.72)	0.04 (174) *p* = 0.971, *d* = 0.00	Exp: 2.28 (422.9), 0.023, 0.21 Comp: 1.95 (395.5), 0.052, 0.19
*Skills: “displays helping skills”	6.58 (2.88)	6.08 (2.67)	3.79 (250) *p* < 0.001, *d* = 0.24	6.10 (2.51)	5.95 (2.55)	1.08 (175) *p* = 0.280, *d* = 0.08	Exp: 1.64 (409.8), 0.101, 0.16 Comp: 0.51 (425), 0.612, 0.05
Intervention Implementation: “knowledge of intervention strategies”	3.98 (3.09)	4.08 (2.91)	−0.61 (247) *p* = 0.544, *d* = −0.04	3.60 (2.52)	3.83 (2.65)	−1.47 (173) *p* = 0.142, *d* = −0.11	Exp: 1.05 (416.5), 0.296, 0.10 Comp: 0.88 (394.7), 0.377, 0.09
Progress Evaluation: “demonstrating knowledge of the assessment of intervention progress and outcome”	3.73 (3.08)	3.66 (2.95)	0.44 (248) *p* = 0.662, *d* = 0.03	3.18 (2.57)	3.36 (2.84)	−1.10 (175) *p* = 0.275, *d* = −0.08	Exp: 1.61 (414.2), 0.108, 0.15 Comp: 1.03 (423), 0.303, 0.10
*SUPERVISION “supervision and training in the professional knowledge base of enhancing and monitoring the professional knowledge of others”	**6.07 (2.42)**	**5.39 (2.46)**	**4.81 (249)** ***p* < 0.001, *d* = 0.30**	**5.53 (2.17)**	**5.07 (2.25)**	**3.67 (176)** ***p* < 0.001, *d* = 0.28**	**Exp: 2.35 (406.7), 0.019, 0.23** **Comp: 1.36 (425), 0.175, 0.13**
Expectations and Roles: “demonstrating knowledge of expectations for supervision”	4.31 (3.36)	4.24 (3.17)	0.41 (248) *p* = 0.682, *d* = 0.03	3.49 (2.68)	3.81 (2.87)	−1.89 (175) *p* = 0.061, *d* = −0.14	Exp: 2.54 (421.1), 0.011, 0.24 Comp: 1.47 (398.0), 0.144, 0.14
*Skills Development: “displaying interpersonal skills of communication and openness to feedback”	7.83 (2.30)	6.51 (2.31)	9.38 (249) *p* < 0.001, *d* = 0.59	7.52 (2.52)	6.31 (2.37)	9.31 (176) *p* < 0.001, *d* = 0.70	Exp: 1.26 (431), 0.207, 0.12 Comp: 0.88 (425), 0.380, 0.09
*INTERDISCIPLINARY SYSTEMS “knowledge of key issues and concepts in related disciplines. Identify and interact with professionals in multiple disciplines”	**6.88 (2.94)**	**6.20 (2.67)**	**4.60 (246)** ***p* < 0.001, *d* = 0.29**	**5.77 (3.09)**	**5.36 (2.80)**	**3.22 (175)** ***p* = 0.002, *d* = 0.24**	**Exp: 3.50 (430), < 0.001, 0.34** **Comp: 3.12 (421), 0.002, 0.31**
*Respectful and Productive Relationships with Individuals from Other Professions: “Demonstrating awareness of the benefits of forming collaborative relationships with other professionals”	6.88 (2.94)	6.20 (2.67)	4.60 (246) *p* < 0.001, *d* = 0.29	5.77 (3.09)	5.36 (2.80)	3.22 (175) *p* = 0.002, *d* = 0.24	Exp: 3.50 (430), <0.001, 0.34 Comp: 3.12 (421), 0.002, 0.31
*MANAGEMENT-ADMINISTRATION “manage the direct delivery of services (DDS) and/or the administration of organizations, programs, or agencies (OPA)”	**7.26 (3.35)**	**6.56 (2.97)**	**4.64 (248)** ***p* < 0.001, *d* = 0.29**	**6.87 (3.55)**	**6.33 (3.24)**	**2.75 (174)** ***p* = 0.007, *d* = 0.21**	**Exp: 1.07 (431), 0.287, 0.10** **Comp: 0.78 (422), 0.437, 0.08**
*Administration: “complying with regulations”	7.26 (3.35)	6.56 (2.97)	4.64 (248) *p* < 0.001, *d* = 0.29	6.87 (3.55)	6.33 (3.24)	2.75 (174) *p* = 0.007, *d* = 0.21	Exp: 1.07 (431), 0.287, 0.10 Comp: 0.78 (422), 0.437, 0.08
*ADVOCACY “actions targeting the impact of social, political, economic or cultural factors to promote change at the individual (client), institutional, and/or systems level”	**6.64 (2.60)**	**6.06 (2.39)**	**3.97 (248)** ***p* < 0.001, *d* = 0.25**	**5.64 (2.70)**	**5.66 (2.42)**	−**0.07 (176)** ***p* = 0.941, *d* =** −**0.01**	**Exp: 3.95 (431), <0.001, 0.39** **Comp: 1.70 (424), 0.090, 0.17**
*Empowerment: “Demonstrating awareness of social, political, economic and cultural factors that impact individuals, institutions and systems that may lead them to seek intervention”	6.64 (2.60)	6.06 (2.39)	3.97 (248) *p* < 0.001, *d* = 0.25	5.64 (2.70)	5.66 (2.42)	−0.07 (176) *p* = 0.941, *d* = −0.01	Exp: 3.95 (431), <0.001, 0.39 Comp: 1.70 (424), 0.090, 0.17

^a^Not included in APA clinical competencies for professional psychology but seems necessary/is an important building block for engaging in evidence-based practice and can be taught at the undergraduate level. Is directly related to the science, research, and EBP areas of competencies.

*Indicates clinical competencies proposed for undergraduates pursuing a career in mental healthcare including writing skills, research methods and critically reviewing research, internship experiences, and education on different mental health professions. Bold value represents the overarching categories.

### 3.2 Q1: clinical competency expectations

#### 3.2.1 Masters programs

Table 2 depicts the mean expectation scores for each overarching clinical competency area and sub-competency. “Moderate to high expectations” (mean ≥ 6.0) are marked with an asterisk. Ten out of 15 overarching competency areas (comprising 27 sub-competencies) met this minimum threshold for expectations from faculty at masters programs: professional values and attitudes (*M* = 8.67), relationships (*M* = 8.14), management-administration (*M* = 7.26), interdisciplinary systems (*M* = 6.88), individual and cultural diversity (6.83), advocacy (*M* = 6.64), reflective practice, self-assessment, and self-care (*M* = 6.55), scientific knowledge and methods (*M* = 6.53), ethical legal standards and policy (*M* = 6.04), and supervision (*M* = 6.07). The sub-competencies in each of these overarching competency areas met the minimum threshold of 6.0 with the following exceptions: “applications based on cultural context” under individual and cultural diversity (*M* = 5.95), “knowledge of ethical, legal, and professional standards and guidelines” (*M* = 5.14) and “awareness and application of ethical decision making” (*M* = 5.44) under ethical legal standards and policy, “self-assessment” (*M* = 5.82) under reflective practice, self-assessment, and self-care, and “expectations and roles” under the supervision category (*M* = 4.31). Conversely, “skills” under the intervention category met the minimum cut-off (*M* = 6.58), although neither the intervention category nor the other sub-competencies in this category did.

#### 3.2.2 Doctoral programs

Eight out of 15 overarching competency areas (comprising 25 sub-competencies) met the minimum threshold for moderate to high expectations from faculty at doctoral-level programs: professional values and attitudes (*M* = 8.78), relationships (*M* = 8.21), scientific knowledge and methods (*M* = 7.40), management-administration (*M* = 6.87), research/evaluation (*M* = 6.50), individual and cultural diversity (*M* = 6.42), reflective practice, self-assessment, and self-care (*M* = 6.22), and ethical legal standards and policy (*M* = 6.04). The sub-competencies in each of these overarching competency areas met the minimum threshold of 6.0 with the exceptions of “applications based on cultural context” (*M* = 5.39) under individual and cultural diversity, “knowledge of ethical, legal and professional standards and guidelines” (*M* = 5.03) and “awareness and application of ethical decision making” (*M* = 5.14) under ethical legal standards and policy, and “reflective practice” (*M* = 5.79) and “self-assessment” (*M* = 5.08) under reflective practice, self-assessment, and self-care. In contrast, “skills” (*M* = 6.10) under intervention and “skills development” (*M* = 7.52) under supervision both met the minimum cut-off.

### 3.3 Q2: masters versus doctoral programs regarding expectations

There were statistically significant (*p* < 0.001) differences between masters and doctoral programs in expectations of competence in seven sub-competencies. Faculty in masters programs expected more competency than faculty in doctoral programs in advocacy, specifically empowerment (*d* = 0.39); in interdisciplinary systems, specifically respectful and productive relationships with individuals from other professions (*d* = 0.34); in reflective practice (*d* = 0.32, a sub-competency of reflective practice, self-assessment, and self-care); and in communication of assessment findings (*d* = 0.31, a sub-competency of assessment). In contrast, faculty in doctoral programs expected more competency than faculty in masters programs in the overarching clinical competency area of scientific knowledge and methods (*d* = −0.38) including the sub-competencies of scientific foundation of psychology (*d* = −0.45) and scientific mindedness (*d* = −0.38). Faculty in doctoral programs also expected more competency in scientific approach to knowledge (*d* = −0.31, a sub-competency of research/evaluation). There were no other statistically significant differences in expectations between masters and doctoral programs.

### 3.4 Q3: expectations versus perceived entry-level competence

#### 3.4.1 Masters programs

Faculty’s expectations of competence were compared to perceived entry-level competence for first-year graduate students in the fifteen overarching competency areas (see Table 2). For masters programs, there were significant differences (*p* ≤ 0.001) between expectations and perceived entry-level competence in 11 of the 15 overarching competency areas assessed; the exceptions were ethical legal standards and policy, reading clinical research, assessment, and intervention. The largest effect sizes were observed in the following competency areas: professional values and attitudes (*d* = 0.72) and relationships (*d* = 0.72) followed by small-to-medium effect sizes in scientific knowledge and methods (*d* = 0.47), individual and cultural diversity (*d* = 0.44), research/evaluation (*d* = 0.36), and reflective practice, self-assessment, and self-care (*d* = 0.34). More specifically, there were statistically significant and medium-to-large differences between faculty expectations and student competence in nine sub-competencies: accountability (*d* = 0.85), deportment (*d* = 0.75), integrity (*d* = 0.73), expressive skills (*d* = 0.72), affective skills (*d* = 0.64), interpersonal relationships (*d* = 0.61), skills development (*d* = 0.59), concern for the welfare of others (*d* = 0.53), and scientific mindedness (*d* = 0.52). In all the differences observed, faculty’s expectations were higher than the perceived entry-level competence of students.

#### 3.4.2 Doctoral programs

For doctoral programs, there were significant differences between expectations and perceived entry-level competence in eight of the 15 overarching competency areas. Effect sizes of these differences were large for professional values and attitudes (*d* = 0.84) and relationships (*d* = 0.84); medium-to-large for scientific knowledge and methods (*d* = 0.77) and research evaluation (*d* = 0.62); and small for reflective practice, self-assessment, and self-care (*d* = 0.32), supervision (*d* = 0.28), evidence-based practice (*d* = 0.26), and individual and cultural diversity (*d* = 0.24). In particular, there were statistically significant and medium-to-large differences between faculty expectations and entry-level competence in 13 sub-competencies: accountability (*d* = 1.07), integrity (*d* = 0.85), scientific mindedness (*d* = 0.76), scientific foundation of psychology (*d* = 0.76), affective skills (*d* = 0.75), expressive skills (*d* = 0.72), interpersonal relationships (*d* = 0.70), skills development (*d* = 0.70) deportment (*d* = 0.68), application of scientific method to practice (*d* = 0.56), concern for the welfare of others (*d* = 0.55), scientific foundation of professional practice (*d* = 0.54), and scientific approach to knowledge generation (*d* = 0.61). In all the differences observed, faculty’s expectations were higher than the perceived entry-level competence of students.

### 3.5 Q4: masters versus doctoral programs regarding perceived entry-level competence

There were statistically significant (*p* < 0.001) differences between masters and doctoral programs in perceived entry-level competence where faculty in doctoral programs rated incoming students as having higher levels of both scientific mindedness (*d* = −0.35, a sub-competency of scientific knowledge and methods) and accountability (*d* = −0.29, a sub-competency of professional values and attitudes) compared to faculty in masters programs. There were no other statistically significant differences in perceived entry-level competence between masters and doctoral programs.

### 3.6 Q5: recommendations for undergraduate psychology departments

Table 3 summarizes the recommendations for undergraduate psychology departments on how to better prepare students to engage in mental healthcare careers. Nine overarching categories were identified: academic skills, general breadth of knowledge, clinical knowledge, cultural awareness and advocacy, graduate training knowledge, interpersonal skills, intrapersonal skills, recommended experiences, and research skills.

**TABLE 3:** Recommendations for Undergraduate Psychology Departments to better prepare students to engage in mental healthcare careers.

	Masters (*n* = 203) n (%)	Doctoral (*n* = 160) n (%)
**ACADEMIC SKILLS**	**24.2%**	**46.3%**
Critical Thinking and Analytical Skills (including logical fallacies)	17 (8.4)	28 (17.5)
Problem-Solving	2 (1.0)	2 (1.3)
Study Skills/Habits and Time Management	2 (1.0)	3 (1.9)
Writing Skills (academic, professional, clinical writing, progress notes; organization; emails; grammar and spelling; APA format and style; give quality feedback on writing)	28 (13.8)	41 (25.6)
**GENERAL BREADTH OF KNOWLEDGE**	**11.8%**	**10.0%**
Broad Curriculum; Broad Perspective	0 (0.0)	5 (3.1)
Courses in other disciplines (family services, social work, business, literature, computer science, political science, music, art, creative expression; interdisciplinary work; philosophy; anthropology)	7 (3.4)	0 (0.0)
Development (child and family development; human developmental stages; neurodevelopment; complexity of human development; attachment theory; social learning theory)	6 (3.0)	1 (0.6)
Read (physical books; in area of interest; textbooks; broadly)	2 (1.0)	2 (1.3)
Theories (application of theories; broader theoretical knowledge; communication theory; feminist; humanistic theories; empirical existential psychology; theories of human behavior; personality theories)	9 (4.4)	8 (5.0)
**CLINICAL KNOWLEDGE**	**32.1%**	**37.0%**
Applied Courses (history of psychology/counseling, intro to counseling, mental health course; intro to school psychology; courses that meet national accreditation guidelines APA, NASP, PCSAS)	4 (2.0)	6 (3.8)
Assessment, Measurement, Psychometrics	5 (2.5)	9 (5.6)
Bridge Research and Theory to Practice (connect academic information with life experiences; connect research to fieldwork; how science compliments helping professions; scientific foundation of mental healthcare)	10 (4.9)	14 (8.8)
Diagnosis and Psychopathology (addiction, suicide; DSM)	5 (2.5)	9 (5.6)
Different Therapy modalities–not just CBT and behaviorism	4 (2.0)	0 (0.0)
Ethics (ethical behavior; ethical decision making; mandatory reporting; boundaries of competence)	13 (6.4)	10 (6.3)
Evidence-Based Practice	9 (4.4)	4 (2.5)
Interventions (treatment planning; knowledge of intervention techniques; case conceptualization skills; crisis interventions; referrals, consultation, short-term solution focused skills, trauma-informed care; person-centered perspectives; indigenous approaches to mental health; common factors of psychotherapeutic change; strengths perspective; behavioral principles training; community health promotion; different theoretical orientations/approaches)	15 (7.4)	7 (4.4)
**CULTURAL AWARENESS AND ADVOCACY**	**36.5%**	**17.6%**
Client/Person in Environment (broader context; global context; client is not just diagnosis; wholistic perspective of person ecological systems theory)	17 (8.4)	2 (1.3)
Cultural Awareness (diversity and cultural influences; multiculturalism; cultural competency; cultural humility; cultural, social, political influences); Diversity, Equity, Inclusion (biases; privilege, racism; oppression; prejudices; marginalization; White supremacy; acknowledging indigenous knowledge systems; awareness of DEI in research, clinical work, and professional practice; critical review of research and theory and whose voices are missing; disabilities; social injustice; teach CRT and Standpoint)	43 (21.2)	19 (11.9)
Intersectionality	4 (2.0)	1 (0.6)
Social Justice	3 (1.5)	3 (1.9)
Structural/Systems Issues and Influences (including family systems)	7 (3.4)	3 (1.9)
**GRADUATE TRAINING KNOWLEDGE**	**19.2%**	**19.4%**
Education on/Exposure to different mental health/helping professions (differences and similarities; degrees; licenses; regulation; approaches, accrediting organizations, etc.; medical vs. wellness model; conceptual frameworks of different professions; understanding of cause of mental health difficulties; non-psychological helping professions; exposure to grad students/professionals in different fields; expectations of different masters programs; exposure to expressive art therapists; helping professions not requiring grad degrees (i.e., case management, DCF settings, inpatient settings); mentorship about differences)	39 (19.2)	24 (15.0)
Graduate School Expectations (not like undergrad)	0 (0.0)	7 (4.4)
**INTERPERSONAL SKILLS**	**41.9%**	**37.5%**
Communication Skills (oral communication; emotional expression)	9 (4.4)	5 (3.1)
Conflict Resolution	3 (1.5)	0 (0.0)
Empathy, Compassion	5 (2.5)	1 (0.6)
Feedback (openness, receptivity, and responsiveness to feedback; application of feedback)	9 (4.4)	12 (7.5)
Helping skills (counseling skills; practice skills; reflective listening; psychological first aid; interviewing skills; being present with client; knowledge that can’t save people and change is slow; non-directive problem solving; not fixing people but understanding situations; reflecting emotion, paraphrasing, empathic confrontation)	21 (10.3)	10 (6.3)
Interpersonal/Relational/Relationship Skills (appropriate interpersonal boundaries; power dynamics; assertiveness; authenticity; vulnerability; humility)	15 (7.4)	9 (5.6)
Professional Behavior (professional socialization; professional development; professional skills; commitment to graduate work; roles; responsibilities; understanding regulations/accountability; professional communication; integrity; honesty; attitude)	16 (7.9)	18 (11.3)
Supervision (understanding supervisory relationship and obligations)	4 (2.0)	4 (2.5)
Teamwork (team-building skills; group dynamics)	3 (1.5)	1 (0.6)
**INTRAPERSONAL SKILLS**	**27.5%**	**16.9%**
Emotional/Mental Wellbeing (emotional skills; emotion regulation; distress tolerance; emotional maturity; personal limitations; comfort with emotions; stress management)	14 (6.9)	4 (2.5)
Psychological Flexibility (openness; openness to being wrong; tolerate ambiguity; growth mindset)	8 (3.9)	8 (5.0)
Self-Awareness and Self-Reflection (introspection; self-exploration)	21 (10.3)	12 (7.5)
Self-Care (active self-care vs. doing nothing; seeking help when needed)	13 (6.4)	3 (1.9)
**RECOMMENDED EXPERIENCES**	**23.1%**	**28.8%**
Internships (volunteering, hands-on experience, field work, informational interviews, practicums, engaged learning experiences; experiential learning in community based agencies; partner with local agencies; service learning experiences; exposure to human suffering; public child welfare/child protective services; prevention experiences; working with culturally diverse, underrepresented, disadvantaged, and clinical, mental-health-related populations)	34 (16.7)	19 (11.9)
Personal Counseling/Therapy (individual and group)	9 (4.4)	2 (1.3)
Research Experiences (work in a research lab; present at a conference; honors thesis; start to finish; independent research)	4 (2.0)	25 (15.6)
**RESEARCH SKILLS**	**17.8%**	**50.6%**
Research Methods/Design; Scientific Process; Scientific Mindset (qualitative and quantitative research methods; limitations of null hypothesis testing; clinical research methods; treatment outcome research; efficacy vs. effectiveness; psychology as a science); Reading, Understanding, and Critiquing Research (finding peer-reviewed sources; critical review of sources; limitations of a single study; conducting a literature review; synthesizing sources; open science; replication crisis)	31 (15.3)	61 (38.1)
Statistics and Math Skills (effect sizes; quantitative reasoning; computing)	5 (2.5)	20 (12.5)
No recommendations (unclear; too general, e.g., foundational courses)	14 (6.9)	4 (2.5)

Bold value represents the overarching categories.

#### 3.6.1 Masters programs

Faculty in masters programs recommended skill development and knowledge in 41 sub-categories with interpersonal skills (41.9%), cultural awareness and advocacy (36.5%), and clinical knowledge (32.1%) generating the most attention overall. More specifically, over one-fifth of respondents (21.2%) recommended more focus on cultural awareness, competency, and humility and equipping students with a better understanding of diversity, equity, and inclusion, including oppression, bias, privilege, racism, classism, homophobia, etc. Nearly one-fifth of respondents (19.2%) recommended educating undergraduates about the different mental healthcare professions including professions that do not require graduate degrees; explaining the differences between psychology, counseling, social work, school psychology, etc.; educating on the differences in approaches to understanding mental health difficulties; and teaching about the state regulations, licensure, degrees, and accrediting organizations. Approximately 17% of faculty (16.7%) recommended internships, including volunteering, service-learning opportunities, informational interviews, as well as specific experiences such as child protective services and local, community-based agencies. Reading and understanding scientific research as well as knowledge in research methods and the scientific process were recommended by 15.3% of faculty. The number one skillset that 13.8% of faculty identified as needing development was writing skills, which included academic, clinical (e.g., progress notes), and professional writing (e.g., emails); APA format; grammar; and spelling.

#### 3.6.2 Doctoral programs

Faculty in doctoral programs recommended skill development and knowledge in 40 sub-categories with research skills (50.6%), interpersonal skills (37.5%), and clinical knowledge (37%) generating the most attention overall. More specifically, a significant portion of the faculty in doctoral programs (38.1%) recommended further training in research methods, the scientific process, clinical research methods, and the science of psychology as well as in reading, understanding, and critiquing research. A quarter of participants (25.6%) highlighted the need for improved writing skills, and 17.5% of faculty recommended the development of critical thinking and analytical skills. Research experiences were encouraged by 15.6% of faculty, and education on the different mental healthcare professions was recommended by 15% of faculty in doctoral programs. Faculty further recommended more attention to statistics and math skills (12.5%).

## 4 Discussion

Our study aimed to identify core clinical competency expectations among graduate-level faculty of incoming, first-year graduate students to guide the development of pre-mental health competency standards for undergraduates. Graduate faculty from masters and doctoral programs across a variety of mental healthcare disciplines were asked to rate their expectations of student competence based on APA’s competency benchmarks in professional psychology (Q1). Overall, faculty of masters and doctoral programs reported moderate to high expectations (mean expectation ≥ 6.0) of first-year graduate students for 11 of 15 overarching competency categories (professional values and attitudes; relationships; management-administration; interdisciplinary systems; individual and cultural diversity; advocacy; reflective practice, self-assessment, and self-care; scientific knowledge and methods; ethical legal standards and policy; supervision; and research/evaluation) comprising 25 sub-competencies.

There were few statistically significant differences in expectations of student competence between masters and doctoral programs (Q2). However, faculty in masters programs reported higher expectations of students than faculty in doctoral programs in four sub-competencies (reflective practice, communication of assessment findings, respectful and productive relationships with individuals from other professions, and empowerment). In contrast, faculty in doctoral programs reported higher expectations of students than faculty in masters programs in three sub-competencies (scientific mindedness, scientific foundation of psychology, and scientific approach to knowledge generation). These findings are consistent with the relative difference between a more practice-oriented degree and a degree that also involves conducting research. Despite these distinctions, the sizes of these differences were medium-to-small (*d*’s ≤ 0.45). Therefore, in order to prepare students flexibly for graduate programs, all sub-competencies that met the minimum expectation threshold by either masters or doctoral program faculty are included in the proposed pre-mental health competency standards. Standardizing expectations may also support undergraduate faculty in preparing students to pursue a range of careers and degrees and in conveying programs’ corresponding expectations.

When comparing expectations to perceived entry-level competence (Q3), faculty in masters and doctoral programs rated their students as not meeting their expectations in the majority of competencies areas, most frequently with medium-to-large differences between expectations and perceived competence. These results suggest that first-year graduate students with bachelor degrees in both masters and doctoral programs are largely failing to meet the clinical competency expectations of their graduate faculty. Furthermore, perceived entry-level competence of students in masters and doctoral programs only differed in two sub-competencies (Q4), scientific mindedness and accountability, where doctoral students were rated as more competent than masters students. Collectively, these findings suggest that first-year graduate students arrive similarly underprepared for both masters and doctoral programs and emphasize the need for pre-mental health standards and related curriculum at the undergraduate level to better prepare students for graduate work in mental healthcare.

To further understand how undergraduate programs can better prepare students to meet the demands of graduate school and a future career in mental healthcare, graduate faculty shared recommendations (Q5). These open-ended responses largely paralleled our quantitative results. Faculty highlighted the need for developing academic skills (e.g., writing skills and critical thinking skills), interpersonal skills (e.g., professional behavior and receptivity to feedback), as well as clinical knowledge including ethical decision-making and connecting research to practice. Respondents recommended that undergraduate programs provide more courses and attention to cultural awareness, competence, and humility as well as diversity, equity, and inclusion. They also recommended participation in internship experiences where students have the opportunity to interact with diverse, under-resourced, and/or clinical populations. In addition, respondents recommended better education on the differences between the mental healthcare professions, their conceptual frameworks, degrees, licenses, etc. to help students select the discipline and degree most suitable for them. Faculty, particularly faculty in doctoral programs, frequently highlighted a need for a better grasp of research methods/design, statistics, and clinical research experience.

In sum, our findings reveal that graduate faculty across a range of mental healthcare disciplines have high expectations of first-year graduate students with bachelor degrees in multiple areas of clinical competence, but there is significant discrepancy between these expectations and perceived student competence. Although several competencies would apply to all undergraduate students (e.g., writing skills, critical thinking skills, communication skills, math skills), our results support the need for undergraduate competency standards that are particularly relevant to pre-mental healthcare (e.g., concern for the welfare of others, interaction of self and others as shaped by cultural diversity and context, and reflective practice) in order to guide colleges and universities in better preparing our future mental health practitioners. Establishing and implementing pre-mental health competencies could improve performance in graduate school and in the workplace as well as facilitate graduate programs in developing important, advanced skillsets instead of providing remedial training in critical areas. The 11 competency areas (and 25 corresponding sub-competencies) that met the minimum expectation score serve as a proposed starting place in the development of pre-mental health competencies (see Table 2). In addition, based on graduate faculty recommendations, writing skills, research methods and critically reviewing research, internship experiences, and education on different mental health professions are added to this list of competencies. Several of these competency areas are included in the guidelines for undergraduate psychology majors (American Psychological Association [APA], 2022), which is particularly helpful for psychology majors; however, many students who pursue careers in mental healthcare are not psychology majors. In addition, there is also significant overlap between our proposed pre-mental health competencies and the competencies proposed by the Council on Social Work Education (CSWE) for master-level social workers. Therefore, we propose these pre-mental health competencies independent of a psychology major and independent of the mental healthcare-related discipline the student intends to pursue. Additional work is necessary to determine whether and how our proposed competencies might be revised for use with undergraduate students (e.g., How might the competencies be assessed? What is the cost—both financial and in terms of teaching power—of addressing each competency? How can we be inclusive and equitable in helping students within and outside of a psychology major achieve these competencies). Furthermore, given that we asked graduate faculty how well prepared they think graduate students are in these competency areas, it will be important to develop assessments of these competencies that are independent of graduate faculty in order to separate the competency being assessed from the assessment method.

### 4.1 Constraints on general applicability

Our study has several limitations. First, only about 5% of faculty who were invited to participate in the survey did so with 2.9% of invited faculty fully completing the survey. We administered the survey during the summer, which could have limited our response rates given that faculty, especially adjunct faculty who comprise ∼50% of all faculty, may not be teaching or checking email during those months. Since we were unable to discern if the faculty identified taught first-year graduate students, it is also possible that a portion of the faculty invited to participate did not complete the survey due to being ineligible. Regardless, our response rate is lower than what could be expected from internet-based surveys (Cook et al., 2000) and suggests that our findings represent a small subset of all graduate faculty and draws into question the representativeness of our sample. Further, it is possible that the faculty who chose to respond were particularly motivated to do so because they have experienced first-year graduate students not being prepared for graduate work. Thus, through self-selection, our results could accentuate the discrepancy between expectations and perceived entry-level of student competence. As this is the first study to our knowledge to explore pre-mental health competencies for undergraduates, we highlight the preliminary nature of these results. Future research should assess expectations and competencies in a more representative sample and with additional stakeholders (e.g., future employers, patients/clients, etc.).

Second, although faculty were invited from a range of mental healthcare professions, a large percentage of faculty were from masters-level social work programs, with some from counseling, but few were from the remaining areas of mental healthcare (e.g., school counseling, school psychology, and marriage and family therapy). Similarly, the majority of faculty from doctoral programs represented clinical psychology programs. Differences between masters and doctoral programs therefore may reflect differences in the disciplines of social work versus clinical psychology. Third, to allow for anonymity, we did not require faculty to report their program, which allowed for the possibility that multiple faculty from the same program participated. Therefore, we are not able to account for the non-independence of our data. This could lead to an overestimation of one program’s perspective over another. However, it is also possible that faculty at the same program could have different perspectives and vantage points on incoming first-year students as well as different recommendations for how to best prepare undergraduate students for graduate training in mental healthcare.

Fourth, although faculty were asked to only rate their incoming, first-year, bachelor-level students, faculty may have reported on all of their first-year graduate students, as it is unlikely that faculty know the degrees of all students. Therefore, the expectations of students may be higher than what can be achieved through undergraduate education alone. Further, it is possible that students entering doctoral programs may already possess a masters degree and have more skills than a student with a bachelor degree, which may explain some of the differences found between the competencies of the students between the two programs.

Fifth, faculty responses about expectations and/or competence might have been influenced by their recollections of particularly strong or weak students, or by the most recent cohort of incoming students, who probably lost critical time in the classroom, internships, jobs, or other relevant settings due to COVID-related disruptions. In either case, responses might not accurately reflect typical first-year graduate students. Indeed, competence ratings were subjective and based on faculty perceptions, rather than measures, which are potentially more objective such as standardized tests or exams. Future research should re-assess expectations and competencies in order to determine if the discrepancies between expectations and competencies are an artifact of COVID-related disruptions or something more stable.

Additionally, another limitation concerns the conclusions that can and cannot be drawn from the qualitative data. Nearly all recommendations on how to better prepare undergraduate students to engage in mental healthcare careers were suggested by a small minority (i.e., 20% or fewer) of respondents. It is unclear whether the remaining respondents explicitly did not view these recommendations as important or whether they simply did not think of them (in which case, one could wonder how critical they really are).

Finally, our clinical competencies were based on the APA’s competency benchmarks and not on competencies or standards put forth by another mental healthcare field. Understandably, this drew criticism from some respondents. More research is needed to determine whether competence in the areas identified would adequately prepare undergraduate students for graduate study and/or employment in a variety of areas—not just clinical psychology and social work. On a related note, although our use of the term “clinical” was intended to reflect any and all mental healthcare professions, some (potential) respondents might not have seen it this way. Indeed, several individuals reported that they did not believe they were eligible for the study or could not participate due to the fact that they were not clinical psychologists. Thus, this term might have prevented a portion of eligible faculty from participating.

Future work in this area should more explicitly highlight commonalities between the different helping professions in order to identify universal competencies that can be targeted at the undergraduate level. In addition, research is needed to develop curriculum recommendations as well as a valid and non-biased assessment with which to measure competence in these areas. In addition to establishing competency standards, it is critical that future research identify steps to support equitable access to achieve these standards. By establishing clear, equitable, measurable, and standardized competencies, the “hidden curriculum” becomes transparent to all students interested in a mental health related career and the competencies can strengthen the care provided and further the wellbeing of our society.

## Data availability statement

The raw data supporting the conclusions of this article will be made available by the authors, without undue reservation.

## Ethics statement

The studies involving humans were approved by the Furman University Institutional Review Board. The studies were conducted in accordance with the local legislation and institutional requirements. The participants provided their written informed consent to participate in this study.

## Author contributions

KB created survey, managed recruitment, collected data, conducted formal quantitative and qualitative data analysis, acquired funds for participant compensation, supervised research assistants, and wrote the original draft of methods, results, and discussion. SW wrote the original draft of introduction. CF was second coder for qualitative data analysis and analyzed interrater reliability for qualitative coding. All authors contributed to conception and study design, significantly contributed to manuscript revision, and read and approved the submitted version.

## Footnotes

1
https://www.apa.org/ed/graduate/benchmarks-guide

## References

[B1] American Academy of Pediatrics, (2021). *AAP-AACAP-CHA declaration of a national emergency in child and adolescent mental health.* Available online at: https://www.aap.org/en/advocacy/child-and-adolescent-healthy-mental-development/aap-aacap-cha-declaration-of-a-national-emergency-in-child-and-adolescent-mental-health/ (accessed March 11, 2023).

[B2] American Association of Medical Colleges [AAMC] (2022). *Core competencies for entering medical students.* Available online at: https://www.aamc.org/services/admissions-lifecycle/competencies-entering-medical-students (accessed March 11, 2023).

[B3] American Psychological Association [APA] (2012). *Competency initiatives in professional psychology.* Available online at: https://www.apa.org/ed/graduate/guide-benchmarks.pdf (accessed March 11, 2023).

[B4] American Psychological Association [APA] (2021). *Careers in psychology.* Available online at: https://www.apa.org/workforce/data-tools/careers-psychology (accessed March 11, 2023).

[B5] American Psychological Association [APA] (2022). *APA guidelines for the undergraduate psychology major version 3.0.* Available online at: https://apps.apa.org/CommentCentral2/attachments/Site97-Undergrad%20Guidelines.pdf (accessed March 11, 2023). 10.1037/a0037562 26866986

[B6] Bingham A. J. Witkowsky P. (2022). “Deductive and inductive approaches to qualitative data analysis,” in *Analyzing and interpreting qualitative data: After the interview*, eds Vanover C. Mihas P. Saldaña J. (Thousand Oaks, CA: SAGE Publications), 133–146.

[B7] Cohen J. (1988). *Statistical power analysis for the behavioral sciences.* New York, NY: Routledge Academic.

[B8] Commission on Accreditation for Marriage and Family Therapy Education [COAMFTE] (2021). *Accreditation Standards: Graduate & Post-Graduate Marriage and Family Therapy Training Programs.* (12.5). Available online at: https://coamfte.org/documents/COAMFTE/Accreditation%20Resources/COAMFTE%20Standards%20Version%2012.5%20-%20Published%20August%202021%20-%208.26.21%20(with%20links).pdf (accessed March 11, 2023).

[B9] Cook C. Heath F. Thompson R. L. (2000). A meta-analysis of response rates in web-or internet-based surveys. *Educ. Psychol. Meas.* 60 821–836.

[B10] Council of University Directors of Clinical Psychology [CUDCP] (2021). *CUDCP preferred predoctoral competencies for clinical psychology.* Available online at: https://cudcp.org/Predoctoral-Competencies (accessed March 11, 2023).

[B11] Council on Social Work Education [CSWE] (2015). *Educational policy and accreditation standards for baccalaureate and master’s social work programs.* Available online at: https://www.uaf.edu/socwork/student-information/checklist/2015%20EPAS%20and%20Glossary.pdf (accessed March 11, 2023).

[B12] Cubic B. Mance J. Turgesen J. N. Lamanna J. D. (2012). Interprofessional education: Preparing psychologists for success in integrated primary care. *J. Clin. Psychol. Med. Sett.* 19 84–92. 10.1007/s10880-011-9291-y 22481240

[B13] De Los Reyes A. Uddin L. Q. (2021). Revising evaluation metrics for graduate admissions and faculty advancement to dismantle privilege. *Nat. Neurosci.* 24 755–758. 10.1038/s41593-021-00836-2 33785908

[B14] Ernestus S. M. Fleming C. J. E. Wenze S. J. Blomquist K. K. (2022). The future of mental health care: Why we need clinical competencies for undergraduate psychology majors. *Behav. Ther.* 45 232–239.

[B15] Godoy L. Hodgkinson S. Robertson H. A. Sham E. Druskin L. Wambach C. G. (2019). Increasing mental health engagement from primary care: The potential role of family navigation. *Pediatrics* 143:e20182418. 10.1542/peds.2018-2418 30877145

[B16] Grus C. L. (2014). “Training, credentialing, and new roles in clinical psychology,” in *The oxford handbook of clinical psychology*, 1st Edn, ed. Barlow D. H. (Oxford: Oxford Handbooks), 10.1093/oxfordhb/9780199328710.013.039

[B17] Hubbard S. (2022). *Ballmer institute sets ambitious goals for oregon’s children.* Eugene, OR: Around the O.

[B18] Kathol R. G. Butler M. McAlpine D. D. Kane R. L. (2010). Barriers to physical and mental condition integrated service delivery. *Psychos. Med.* 72 511–518. 10.1097/PSY.0b013e3181e2c4a0 20498293

[B19] Krippendorff K. (2004). Measuring the reliability of qualitative text analysis data. *Qual. Quant.* 38 787–800.

[B20] Landrum R. E. Harrold R. (2003). What employers want from psychology graduates. *Teach. Psychol.* 30 131–133.

[B21] Marques L. Bartuska A. D. Cohen J. N. Youn S. J. (2020). Three steps to flatten the mental health need curve amid the COVID-19 pandemic. *Depress. Anxiety* 37 405–406. 10.1002/da.23031 32429005 PMC7272919

[B22] Naufel K. Z. Appleby D. C. Young J. Van Kirk J. F. Spencer S. M. Rudmann J. (2018). *The skillful psychology student: Prepared for success in the 21st century workplace.* Available online at: https://www.apa.org/careers/resources/guides/transferable-skills.pdf (accessed March 11, 2023).

[B23] Raviola G. Naslund J. A. Smith S. L. Patel V. (2019). Innovative models in mental health delivery systems: Task sharing care with non-specialist providers to close the mental health treatment gap. *Curr. Psychiatry Rep.* 21:44. 10.1007/s11920-019-1028-x 31041554

[B24] Sammons M. T. (2022). Unmet mental health needs associated with COVID must be addressed. *J. Health Servic. Psychol.* 48 1–2. 10.1007/s42843-022-00057-6 35128462 PMC8809244

[B25] Sanderson W. C. Arunagiri V. Funk A. P. Ginsburg K. L. Krychiw J. K. Limowski A. R. (2020). The nature and treatment of pandemic-related psychological distress. *J. Contemp. Psychother.* 50 251–263. 10.1007/s10879-020-09463-7 32836377 PMC7320243

[B26] The World Mental Health Survey Consortium (2004). Prevalence, severity, and unmet need for treatment of mental disorders in the World Health Organization World Mental Health Surveys. *JAMA* 291 2581–2590. 10.1001/jama.291.21.2581 15173149

[B27] Vears D. F. Gillam L. (2022). Inductive content analysis: A guide for beginning qualitative researchers. *Focus Health Profess. Educ. Multi Discipl. J.* 23 111–127.

