# Supplementary material for: Assessing the need for pre-mental health competencies in undergraduate education: insights from graduate faculty surveys
Source: Front Psychol. 2024 Jan 5;14:1252451. doi: 10.3389/fpsyg.2023.1252451 (PMC10797007; doi:10.3389/fpsyg.2023.1252451)
Supplement: Supplementary file 1 [file Data_Sheet_1.PDF]

Supplemental Table 1: Accrediting Agencies

Field	Accrediting Agency	
Clinical Psychology	• American Psychological Association (APA)<br>• Psychological Clinical Science Accreditation System (PCSAS)	• https://www.accreditation.apa.org/accredited-programs<br>• https://www.pcsas.org/accreditation/accredited-programs/
Counseling Psychology	• Council for Accreditation of Counseling and Related Educational Programs (CACREP)	• https://www.cacrep.org/directory/
Social Work	• Council on Social Work Education (CSWE)	• https://www.cswe.org/accreditation/
Marriage and Family Therapy	• Commission on Accreditation for Marriage and Family Therapy Education (COAMFTE)<br>• International Accreditation Commission for Systemic Therapy Education (IACSTE)	• https://coamfte.org/COAMFTE/Directory_of_Accredited_Programs/MFT_Training_Programs.aspx<br>• https://www.ifta-familytherapy.org/approvedprograms.php
School Psychology	• National Association for School Psychology (NASP)	• https://www.nasponline.org/standards-and-certification/graduate-program-approval-and-accreditation/program-approval/approved-programs
Other websites consulted	• National Council for Accreditation of Teacher Education (NCATE)<br>• Northwest Commission on Colleges and Universities (NWCCU)<br>• Western Association of Schools and Colleges (WASC) Senior College and University Commission (WSCUC)	• NCATE: https://www.chea.org/national-council-accreditation-teacher-education<br>• NWCCU: https://nwccu.org/member-institutions/directory/<br>• WSCUC: https://www.wscuc.org/directory/

Supplemental Appendix A. Outreach Email & Survey

Hello Professor X,

My colleagues and I are clinical psychology professors at undergraduate institutions. We believe that students' undergraduate education can play a critical role in preparing those who pursue a career in mental health care.

With this 15-minute survey, we seek to determine what graduate faculty believe is needed at the undergraduate level to better prepare future providers. This survey asks you about the strengths and weaknesses of your first-year graduate students--what you wish students had a better grasp of and what students come in already prepared to do.

Our questions are derived from APA's Competency Benchmarks in Professional Psychology, for which there are standards for determining readiness for clinical practicum, internship, and practice.

The data we collect will only be reported in aggregate and will not be connected to your name, university, or program.

Thank you, in advance, for taking the time to complete this survey! We appreciate you sharing this study with other faculty, where appropriate.

Follow this link to the Survey: X

Or copy and paste the URL below into your internet browser: X

Follow the link to opt out of future emails: X

Sincerely,

X

Survey

1. **Pick <u>one</u> program in which you teach first-year graduate students (those with Bachelor's degrees only) and indicate the program's degree (MA, MSW, MFT, MEd, PhD, PsyD, etc.) and field of study (clinical, counseling, social work, school counseling, marital and family therapy). Please select a terminal masters program over a doctoral program if you teach in both. You will be answering questions based on the program you indicate below.**
   Name of selected program (e.g., X University MA in Clinical Mental Health Counseling):

   ____

2. Indicate degree of selected program:

   ▼ MA (1) ... Other (7)

   Options included: MA, MSW, MFT, Med, PsyD, PhD, Other

3. If other degree, please specify:

   ____

4. Indicate field of study of selected program:

   ▼ Clinical (1) ... Other (6)

   Options included: Clinical, Counseling, Social Work, School Counseling, School Psychology, Marital and Family Therapy, Other

5. If other field, please specify:

   ____

6. **How many years have you taught students in this program?**
   1-2 years (1)
   3-5 years (2)
   6+ years (3)

---

7. **What is your highest degree?**

   ▼ MA (1) ... Other (7)

---

8. If other degree, please specify:

   ____

Based on the program you indicated previously, please rate your **average** **first-year graduate student**. **Rate** only those with bachelor degrees--not those with additional graduate experience--on the following:

(1) the degree to which you **EXPECT** competence in the following areas **at the start of their graduate program** on a scale from 0 (*do not expect competence in this area*) to 10 (*expect high competence in this area*)

(2) the degree to which they **HAVE** competence, on average, in the following areas **at the start of their graduate program** on a scale from 0 (*no competence in this area*) to 10 (*extremely competent in this area).*

---

9. These questions assess competence in areas related to "Professional Values and Attitudes" defined as "behavior and comportment that reflect the values and attitudes of psychology." Please rate the competence level of incoming first-year students at the start of graduate school--those with only a Bachelor's degree and no additional training.

	Expectations of Competence	Competence Level
Integrity: "honesty, personal responsibility and adherence to professional values" (1)	▼ 0-do not expect competence in this (1 ... N/A (no basis for judgment) (12)	▼ 0-no competence in this (1 ... N/A (no basis for judgment) (12)
Deportment: "conducting oneself in a professional manner" (2)	▼ 0-do not expect competence in this (1 ... N/A (no basis for judgment) (12)	▼ 0-no competence in this (1 ... N/A (no basis for judgment) (12)
Accountability: "accountable and reliable" (3)	▼ 0-do not expect competence in this (1 ... N/A (no basis for judgment) (12)	▼ 0-no competence in this (1 ... N/A (no basis for judgment) (12)
Concern for the Welfare of Others: "demonstrating awareness of the need to uphold and protect the welfare of others" (4)	▼ 0-do not expect competence in this (1 ... N/A (no basis for judgment) (12)	▼ 0-no competence in this (1 ... N/A (no basis for judgment) (12)
Professional Identity: "demonstrating an understanding of self as professional; thinking like a clinician" (5)	▼ 0-do not expect competence in this (1 ... N/A (no basis for judgment) (12)	▼ 0-no competence in this (1 ... N/A (no basis for judgment) (12)

10. These questions assess competence in areas related to **“Individual and Cultural Diversity”** defined as “awareness, sensitivity and skills in working professionally with diverse individuals, groups, and communities who represent various cultural and personal background and characteristics defined broadly and consistent with APA policy.” **Please rate the competence level of incoming first-year students at the start of graduate school--those with only a Bachelor's degree and no additional training.**

	Expectations of Competence	Competence Level
Self as Shaped by Cultural Diversity: “demonstrating knowledge, awareness, and understanding of one’s own dimensions of diversity and attitudes towards diverse others” (1)	▼ 0-do not expect competence in this (1 ... N/A (no basis for judgment) (12)	▼ 0-no competence in this (1 ... N/A (no basis for judgment) (12)
Others as Shaped by Cultural Diversity: “demonstrating knowledge, awareness, and understanding of other individuals as cultural beings” (2)	▼ 0-do not expect competence in this (1 ... N/A (no basis for judgment) (12)	▼ 0-no competence in this (1 ... N/A (no basis for judgment) (12)
Interaction of Self and Others as Shaped by Cultural Diversity and Context: “demonstrating knowledge, awareness, and understanding of interactions between self and diverse others“ (3)	▼ 0-do not expect competence in this (1 ... N/A (no basis for judgment) (12)	▼ 0-no competence in this (1 ... N/A (no basis for judgment) (12)
Applications based on Cultural Context: “demonstrating knowledge of and sensitivity to the scientific, theoretical, and contextual issues related to individual and cultural diversity as they apply to assessment, treatment, research, relationships, etc..” (4)	▼ 0-do not expect competence in this (1 ... N/A (no basis for judgment) (12)	▼ 0-no competence in this (1 ... N/A (no basis for judgment) (12)

11. These questions assess competence in areas related to **“Ethical Legal Standards and Policy”** defined as “application of ethical concepts and awareness of legal issues regarding professional activities with individuals, groups, and organizations.” **Please rate the competence level of incoming first-year students at the start of graduate school--those with only a Bachelor's degree and no additional training.**

	Expectations of Competence	Competence Level
Knowledge of ethical, legal and professional standards and guidelines: “demonstrating knowledge of the principles of the APA Ethical Principles and Code of Conduct” (1)	▼ 0-do not expect competence in this (1 ... N/A (no basis for judgment) (12)	▼ 0-no competence in this (1 ... N/A (no basis for judgment) (12)
Awareness and Application of Ethical Decision Making: “demonstrating awareness of the importance of applying an ethical decision model to practice” (2)	▼ 0-do not expect competence in this (1 ... N/A (no basis for judgment) (12)	▼ 0-no competence in this (1 ... N/A (no basis for judgment) (12)
Ethical Conduct: “displaying ethical attitudes and values” (3)	▼ 0-do not expect competence in this (1 ... N/A (no basis for judgment) (12)	▼ 0-no competence in this (1 ... N/A (no basis for judgment) (12)
Reflective Practice: “Displaying mindfulness and self-awareness; engaging in reflection regarding professional practice” (4)	▼ 0-do not expect competence in this (1 ... N/A (no basis for judgment) (12)	▼ 0-no competence in this (1 ... N/A (no basis for judgment) (12)
Self-Assessment: “Demonstrating knowledge of core competencies; engaging in initial self-assessment re: competencies” (5)	▼ 0-do not expect competence in this (1 ... N/A (no basis for judgment) (12)	▼ 0-no competence in this (1 ... N/A (no basis for judgment) (12)
Self-Care: “attention to personal health and well-being to assure effective professional functioning” (6)	▼ 0-do not expect competence in this (1 ... N/A (no basis for judgment) (12)	▼ 0-no competence in this (1 ... N/A (no basis for judgment) (12)
Participation in Supervision Process: “demonstrating straightforward, truthful, and respective communication in supervisory relationship” (7)	▼ 0-do not expect competence in this (1 ... N/A (no basis for judgment) (12)	▼ 0-no competence in this (1 ... N/A (no basis for judgment) (12)

12. These questions assess competence in areas related to **“Relationships”** defined as "relating effectively and meaningfully with individuals, groups, and/or communities.” **Please rate the competence level of incoming first-year students at the start of graduate school--those with only a Bachelor's degree and no additional training.**

	Expectations of Competence	Competence Level
Interpersonal Relationships: “displaying interpersonal skills; forming and maintaining productive and respectful relationships with clients, peers/colleagues, supervisors” (1)	▼ 0-do not expect competence in this (1 ... N/A (no basis for judgment) (12)	▼ 0-no competence in this (1 ... N/A (no basis for judgment) (12)
Affective Skills: “displaying affective skills; Negotiating differences and handling conflict; providing effective feedback to others and receiving feedback nondefensively” (2)	▼ 0-do not expect competence in this (1 ... N/A (no basis for judgment) (12)	▼ 0-no competence in this (1 ... N/A (no basis for judgment) (12)
Expressive Skills: “communicates ideas, feelings, and information clearly using verbal, nonverbal, and written skills” (3)	▼ 0-do not expect competence in this (1 ... N/A (no basis for judgment) (12)	▼ 0-no competence in this (1 ... N/A (no basis for judgment) (12)

13. These questions assess competence in areas related to **“Scientific Knowledge and Methods"** defined as “understanding of research, research methodology, techniques of data collection and analysis, biological bases of behavior, cognitive affective bases of behavior, and development across the lifespan. Respect for scientifically-derived knowledge.” **Please rate the competence level of incoming first-year students at the start of graduate school--those with only a Bachelor's degree and no additional training.**

	Expectations of Competence	Competence Level
Scientific Mindedness: "displaying critical scientific thinking; Valuing and applying scientific methods to professional practice" (1)	▼ 0-do not expect competence in this (1 ... N/A (no basis for judgment) (12)	▼ 0-no competence in this (1 ... N/A (no basis for judgment) (12)
Scientific Foundation of Psychology: “demonstrating an understanding of psychology as a science” (2)	▼ 0-do not expect competence in this (1 ... N/A (no basis for judgment) (12)	▼ 0-no competence in this (1 ... N/A (no basis for judgment) (12)
Scientific Foundation of Professional Practice: “understanding the scientific foundation of professional practice” (3)	▼ 0-do not expect competence in this (1 ... N/A (no basis for judgment) (12)	▼ 0-no competence in this (1 ... N/A (no basis for judgment) (12)

14. These questions assess competence in areas related to **“Research/Evaluation”** defined as “generating research that contributes to the professional knowledge base and/or evaluates the effectiveness of various professional activities.” **Please rate the competence level of incoming first-year students at the start of graduate school--those with only a Bachelor's degree and no additional training.**

	Expectations of Competence	Competence Level
Scientific Approach to Knowledge Generation: “demonstrating skills and habits in seeking, applying, and evaluating theoretical and research knowledge relevant to the practice of psychology” (1)	▼ 0-do not expect competence in this (1 ... N/A (no basis for judgment) (12)	▼ 0-no competence in this (1 ... N/A (no basis of judgment) (12)
Application of Scientific Method to Practice: “demonstrating knowledge of application of scientific methods to evaluating practices, interventions, and programs” (2)	▼ 0-do not expect competence in this (1 ... N/A (no basis for judgment) (12)	▼ 0-no competence in this (1 ... N/A (no basis of judgment) (12)

15. This question assesses competence related to **“Evidence-Based Practice”** defined as “integration of research and clinical expertise in the context of patient factors.” **Please rate the competence level of incoming first-year students at the start of graduate school--those with only a Bachelor's degree and no additional training.**

	Expectations of Competence	Competence Level
Knowledge and Application of Evidence-Based Practice: “demonstrating knowledge of scientific, theoretical, and contextual bases of assessment and intervention; demonstrating knowledge of the value of evidence-based practice" (1)	▼ 0-do not expect competence in this (1 ... N/A (no basis of judgment) (12)	▼ 0-no competence in this (1 ... N/A (no basis for judgment) (12)

16. These questions assess competence in areas related to **"Assessment"** defined as "assessment and diagnosis of problems, capabilities, and issues associated with individuals, groups, and/or communities." **Please rate the competence level of incoming first-year students at the start of graduate school--those with only a Bachelor's degree and no additional training.**

	Expectations of Competence	Competence Level
Knowledge of Measurement and Psychometrics: "Demonstrating knowledge of the scientific, theoretical, and contextual basis of test construction and interviewing" (1)	▼ 0-do not expect competence in this (1 ... N/A (no basis for judgment) (12)	▼ 0-no competence in this (1 ... N/A (no basis for judgment) (12)
Knowledge of Assessment Methods: "Demonstrating knowledge of administration and assessment scoring, including clinical interviewing and mental status exam" (2)	▼ 0-do not expect competence in this (1 ... N/A (no basis for judgment) (12)	▼ 0-no competence in this (1 ... N/A (no basis for judgment) (12)
Application of Assessment Methods: "Demonstrating knowledge of measurement across domains of functioning and practice settings" (3)	▼ 0-do not expect competence in this (1 ... N/A (no basis for judgment) (12)	▼ 0-no competence in this (1 ... N/A (no basis for judgment) (12)
Diagnosis: "Demonstrating knowledge regarding the range of normal/abnormal behavior in the context of human development and diversity" (4)	▼ 0-do not expect competence in this (1 ... N/A (no basis for judgment) (12)	▼ 0-no competence in this (1 ... N/A (no basis for judgment) (12)
Conceptualization: "Demonstrating knowledge of formulating diagnosis and case conceptualization" (5)	▼ 0-do not expect competence in this (1 ... N/A (no basis for judgment) (12)	▼ 0-no competence in this (1 ... N/A (no basis for judgment) (12)
Communication of Assessment Findings: "Demonstrating awareness of models of report writing and progress notes" (6)	▼ 0-do not expect competence in this (1 ... N/A (no basis for judgment) (12)	▼ 0-no competence in this (1 ... N/A (no basis for judgment) (12)

17. These questions assess competence in areas related to **"Intervention"** defined as "interventions designed to alleviate suffering and to promote health and well-being of individuals, groups, and/or communities." **Please rate the competence level of incoming first-year students at the start of graduate school--those with only a Bachelor's degree and no additional training.**

	Expectations of Competence	Competence Level
Intervention Planning: "understanding the relationship between assessment and intervention" (1)	▼ 0-do not expect competence in this (1 ... N/A (no basis for judgment) (12)	▼ 0-no competence in this (1 ... N/A (no basis for judgment) (12)
Skills: "displays helping skills" (2)	▼ 0-do not expect competence in this (1 ... N/A (no basis for judgment) (12)	▼ 0-no competence in this (1 ... N/A (no basis for judgment) (12)
Intervention Implementation: "knowledge of intervention strategies" (3)	▼ 0-do not expect competence in this (1 ... N/A (no basis for judgment) (12)	▼ 0-no competence in this (1 ... N/A (no basis for judgment) (12)
Progress Evaluation: "demonstrating knowledge of the assessment of intervention progress and outcome" (4)	▼ 0-do not expect competence in this (1 ... N/A (no basis for judgment) (12)	▼ 0-no competence in this (1 ... N/A (no basis for judgment) (12)

18. These questions assess competence in areas related to **"Supervision"** defined as "supervision and training in the professional knowledge base of enhancing and monitoring the professional knowledge of others." **Please rate the competence level of incoming first-year students at the start of graduate school--those with only a Bachelor's degree and no additional training.**

	Expectations of Competence	Competence Level
Expectations and Roles: "demonstrating knowledge of expectations for supervision" (1)	▼ 0-do not expect competence in this (1 ... N/A (no basis for judgment) (12)	▼ 0-no competence in this (1 ... N/A (no basis for judgment) (12)
Skills Development: "displaying interpersonal skills of communication and openness to feedback" (13)	▼ 0-do not expect competence in this (1 ... N/A (no basis for judgment) (12)	▼ 0-no competence in this (1 ... N/A (no basis for judgment) (12)

19. These question assesses competence in areas related to **“Interdisciplinary Systems”** defined as “knowledge of key issues and concepts in related disciplines. Identify and interact with professionals in multiple disciplines.” **Please rate the competence level of incoming first-year students at the start of graduate school--those with only a Bachelor's degree and no additional training.**

	Expectations of Competence	Competence Level
Respectful and Productive Relationships with Individuals from Other Professions: “Demonstrating awareness of the benefits of forming collaborative relationships with other professionals” (2)	▼ 0-do not expect competence in this (1 ... N/A (no basis for judgment) (12)	▼ 0-no competence in this (1 ... N/A (no basis for judgment) (12)

20. This question assesses competence related to **“Management-Administration”** defined as “manage the direct delivery of services (DDS) and/or the administration of organizations, programs, or agencies (OPA).” **Please rate the competence level of incoming first-year students at the start of graduate school--those with only a Bachelor's degree and no additional training.**

	Expectations of Competence	Competence Level
Administration: “complying with regulations” (1)	▼ 0-do not expect competence in this (1 ... N/A (no basis for judgment) (12)	▼ 0-no competence in this (1 ... N/A (no basis for judgment) (12)

21. The following question assesses competence in areas related to **“Advocacy”** defined as “actions targeting the impact of social, political, economic or cultural factors to promote change at the individual (client), institutional, and/or systems level.” **Please rate the competence level of incoming first-year students at the start of graduate school--those with only a Bachelor's degree and no additional training.**

	Expectations of Competence	Competence Level
Empowerment: “Demonstrating awareness of social, political, economic and cultural factors that impact individuals, institutions and systems that may lead them to seek intervention” (1)	▼ 0-do not expect competence in this (1 ... N/A (no basis for judgment) (12)	▼ 0-no competence in this (1 ... N/A (no basis for judgment) (12)

22. The following questions assess competence in **reading, understanding, and interpreting clinical research. Please rate the competence level of incoming first-year students at the start of graduate school--those with only a Bachelor's degree and no additional training.**

	Expectations of Competence	Competence Level
Randomized Controlled Trials: “Demonstrating an understanding of study design, methodological limitations, statistical interpretation, and clinical implications of findings of RCTs.” (1)	▼ 0-do not expect competence in this (1 ... N/A (no basis for judgment) (12)	▼ 0-no competence in this (1 ... N/A (no basis for judgment) (12)
Meta-Analyses: “Demonstrating an understanding of study design, methodological limitations, statistical interpretation, and clinical implications of findings of meta-analyses.” (3)	▼ 0-do not expect competence in this (1 ... N/A (no basis for judgment) (12)	▼ 0-no competence in this (1 ... N/A (no basis for judgment) (12)

23. What would you recommend to undergraduate psychology departments to better prepare students to engage in their mental health care careers?

____

____

____

____

____

24. What courses should students take, what topics should they cover, and/or what skills would be helpful for students to develop during their undergraduate years to better prepare them to engage in their mental health care careers?

____

____

____

____

____

25. Do you teach evidence-based practice?

○ Yes (4)

○ No (5)

○ I don't know (6)

26. Do you teach students how to read and evaluate RCTs and meta-analyses?

- ○ Yes (4)
- ○ No (5)
- ○ I don't know (6)

---

27. What empirically-supported treatments do you teach?

____

____

____

____

____

---

28. Through your program and external partners, which empirically-supported treatments do your students have an opportunity to train in?

____

____

____

____

____

---

29. What is the average age range of incoming students in your program?

____
